# A Transcriptomic Signature of Mouse Liver Progenitor Cells

**DOI:** 10.1155/2016/5702873

**Published:** 2016-10-03

**Authors:** Adam M. Passman, Jasmine Low, Roslyn London, Janina E. E. Tirnitz-Parker, Atsushi Miyajima, Minoru Tanaka, Helene Strick-Marchand, Gretchen J. Darlington, Megan Finch-Edmondson, Scott Ochsner, Cornelia Zhu, James Whelan, Bernard A. Callus, George C. T. Yeoh

**Affiliations:** ^1^School of Chemistry and Biochemistry, The University of Western Australia, Crawley, WA 6009, Australia; ^2^The Centre for Medical Research, Harry Perkins Institute of Medical Research, Nedlands, WA 6009, Australia; ^3^ARC Centre of Excellence in Plant Energy Biology, The University of Western Australia, Crawley, WA 6009, Australia; ^4^School of Biomedical Sciences, Curtin Health Innovation Research Institute, Curtin University, Bentley, WA 6102, Australia; ^5^School of Medicine and Pharmacology, The University of Western Australia, Fremantle, WA 6160, Australia; ^6^Institute of Molecular and Cellular Biosciences, The University of Tokyo, Tokyo 113-8654, Japan; ^7^Unité de Génétique de la Différenciation, Institut Pasteur, 75015 Paris, France; ^8^Huffington Center on Aging, Baylor College of Medicine, Houston, TX 77030, USA; ^9^Department of Physiology, NUS Yong Loo Lin School of Medicine, Singapore 117411; ^10^Mechanobiology Institute (MBI), National University of Singapore, Singapore 117411; ^11^Department of Molecular and Cellular Biology, Baylor College of Medicine, Houston, TX 77030, USA; ^12^Department of Animal, Plant and Soil Sciences, La Trobe University, Melbourne, VIC 3086, Australia; ^13^School of Health Sciences, The University of Notre Dame Australia, Fremantle, WA 6959, Australia

## Abstract

Liver progenitor cells (LPCs) can proliferate extensively, are able to differentiate into hepatocytes and cholangiocytes, and contribute to liver regeneration. The presence of LPCs, however, often accompanies liver disease and hepatocellular carcinoma (HCC), indicating that they may be a cancer stem cell. Understanding LPC biology and establishing a sensitive, rapid, and reliable method to detect their presence in the liver will assist diagnosis and facilitate monitoring of treatment outcomes in patients with liver pathologies. A transcriptomic meta-analysis of over 400 microarrays was undertaken to compare LPC lines against datasets of muscle and embryonic stem cell lines, embryonic and developed liver (DL), and HCC. Three gene clusters distinguishing LPCs from other liver cell types were identified. Pathways overrepresented in these clusters denote the proliferative nature of LPCs and their association with HCC. Our analysis also revealed 26 novel markers, LPC markers, including* Mcm2* and* Ltbp3*, and eight known LPC markers, including* M2pk* and* Ncam*. These markers specified the presence of LPCs in pathological liver tissue by qPCR and correlated with LPC abundance determined using immunohistochemistry. These results showcase the value of global transcript profiling to identify pathways and markers that may be used to detect LPCs in injured or diseased liver.

## 1. Introduction

Liver progenitor cells (LPCs) have garnered substantial interest in the field of liver biology due to their enormous regenerative capacity [1, 2] which positions LPCs as a strong candidate for cell therapy to treat liver disease. Whilst their link to liver disease [3–5] and potential as a liver cancer stem cell constrain their utility, they afford a model to investigate the molecular and cellular mechanisms that underlie tumorigenic transformation.

In many chronic liver diseases, when proliferation of hepatocytes is limited, LPCs replicate and differentiate, providing an alternate source of hepatocytes needed for regeneration [6]. This “LPC response” is observed in humans in cases of alcoholic liver disease, hemochromatosis, hepatitis C and B infection, and HCC [4, 5, 7–9]. Other liver pathologies may also involve expansion of LPCs; however, their role in these instances is undefined [10]. A rodent model of chronic liver disease, the choline-deficient, ethionine-supplemented (CDE) diet which results in fatty liver and subsequently fibrosis and cirrhosis, induces LPCs in rats [11] and mice [12] and has been used extensively for studying LPCs. This model was used to reveal Wnt and Notch-controlled signaling that specifies LPCs to the hepatocytic and cholangiocytic lineages, respectively [13]. Other strategies to induce LPCs include administration of 3,5-diethoxy-carbonyl-1,4-dihydrocollidine (DDC) [11] and acetyl aminofluorene to block hepatocyte proliferation followed by partial hepatectomy [14] and administration of alkylating agents, monocrotaline [15] or retrorsine [16].

The degree to which LPCs contribute to liver regeneration is controversial. In contrast to several publications that specify a role for LPCs in liver regeneration [6, 17, 18], a recent review [19] highlighted lineage-tracing studies [20, 21] in which LPCs did not. This discordance may be dependent on the method used to induce liver pathology. Nevertheless, using different approaches, several laboratories have established LPC lines that are capable of* in vitro* differentiation into cholangiocytes and hepatocytes [22–25].

LPCs are a heterogeneous cell population and whether LPCs induced by different etiologies and via various models are identical or even share similar genetic profiles is unknown. Comparing lines generated in different laboratories thus mandates caution when interpreting findings to reach generalized conclusions on the biology of LPCs. These issues are compounded as current LPC markers often stain cholangiocytes as well, highlighting a requirement for additional markers.

Proliferation of LPCs and development of liver cancer have been correlated. In mice, inducible liver-specific expression of c-myelocytomatosis (c-Myc) oncogene stimulated proliferation of an immature cell population resulting in HCC [26]. Upon c-Myc inactivation, tumor cells differentiated into hepatocytes and cholangiocytes, suggesting the HCCs formed from LPC-like cells. Fibrolamellar HCCs are enriched with cancer stem cells resembling “biliary tree stem cells,” LPC precursors [27]. In another study, comprehensive gene array analysis of human HCC identified gene amplification of yes-associate protein (YAP) and cellular inhibitor of apoptosis (cIAP1) as potential oncogenes [28]. In the same study, overexpression of cIAP1 in a p53 null LPC cell line substantially reduced tumor onset time and increased tumor burden. Additional links between the Hippo pathway, LPCs, and HCC development have been identified [29–31]. High YAP expression marks progenitor cells within the liver and expression within hepatocytes is sufficient to differentiate them into LPC-like cell [32]. This may have implications for HCC development, especially for tumors described as poorly differentiated. It is therefore of interest to obtain a transcript profile of LPCs, both to aid discovery of markers for detection and to further study their association with HCC. We hypothesize that LPCs will display a transcript profile that reflects some, if not many, of the “hallmarks” of cancer, in particular, dysregulated cell proliferation and resistance to cell death [33].

In this study, we performed transcriptome analysis of established and well-characterized LPC lines and pooled LPC transcriptome data from many laboratories to determine whether LPCs isolated by different groups using different approaches are similar or distinguishable based on their transcriptome. Additionally, we interrogated several repositories of transcriptome data for comparison with other cell and tissue types including datasets of muscle and embryonic stem cell lines, embryonic and developed liver, and HCC. We identified signaling pathways and promoters that are consistently active in LPC lines to provide greater insight into their biology. Lastly, we identified novel LPC markers and validated their utility to identify LPCs in different mouse models of liver pathology.

## 2. Materials and Methods

### 2.1. Capturing the Transcriptomes

The LPC lines were derived by different procedures in multiple laboratories. Bipotential murine embryonic liver (BMEL) cell lines were derived from CBA/J × C57Bl/6J cross embryonic livers at 14 days post coitum. Bipotential murine oval liver (BMOL) cell lines were derived from 7-week-old male C57BL/6 strain wild-type mice livers subjected to 3 weeks of CDE diet. Tokyo-LPC (T-LPC) was isolated by selection of EPCAM+ cells from DDC-injured livers. The p53-immortalized liver (PIL) 1, 2, 3, 4, and 5 lines were derived from adult p53 null (C57BL/6 strain) mice. These LPC lines have been characterized previously [22, 23, 25, 34, 35], by morphology, bipotentiality, proliferative ability, and expression of LPC markers. For microarrays generated in our laboratory, LPC RNA was isolated using TRIzol (Invitrogen) and RNeasy mini kit (Qiagen) with DNase I treatment following manufacturer's recommendations. RNA quality was verified using an Agilent 2100 Bioanalyser. Synthesis and labeling of cDNA and cRNA, fragmentation, hybridization, washing, staining, and scanning of the Affymetrix Mouse Genome 430 2.0 GeneChips including quality control checks were performed according to the manufacturer's instructions.

### 2.2. Normalization of Microarray Experiments

Microarray experiments (381) that fulfilled the requirements of having at least three replicates performed on the Affymetrix Mouse Genome 430 2.0 or 430A GeneChips platforms were downloaded from the National Centre for Biotechnology Information Gene Expression Omnibus (GEO) Repository. Datasets included those generated from LPC lines, C2C12 muscle cell lines, embryonic stem cells, DL, HCC, and embryonic liver. We performed a meta-analysis of 405 microarrays comprising the 381 publically available microarrays and 24 LPC microarrays generated by our laboratory, using the BMOL, T-LPC, and PIL1-5 LPC lines. The arrays generated from these seven LPC lines have been uploaded to the GEO (GSE85114). In addition to these seven LPC lines, our meta-analysis included three separate, publically sourced BMEL lines. A summary of details pertaining to all arrays used in this manuscript is included in Supplementary Table 1 in Supplementary Material available online at http://dx.doi.org/10.1155/2016/5702873.

Probe sets from all 405 arrays were filtered to include only present sets since the proportion of absent ones can considerably affect the data median. Presence for a sample was established following MAS (MicroArray Suite) 5.0 summarization (*p* value < 0.05) when the probe set was present in at least 75% of replicates. The median gene expression and Median Absolute Deviation (MAD) were determined for each microarray, as a representation of the spread of the data. Median standardization of data involved subtracting the median array expression from the expression of each probe set and then dividing the result by the MAD of each microarray. This produces a median of 0 for each microarray. Values were then multiplied by 500 to increase the spread and standardize to a common spread that is within the observed range of MAD for most of the microarrays. Finally, a constant of 750 was added to the values to increase the median to 750, also within the observed range of medians.

As the Mouse Genome 430 2.0 GeneChip contains more probes than the 430A GeneChip, the dataset was truncated to include only the 430A probes. log⁡2 transformation and quantile normalization were performed to standardize the distribution of the probe set intensities to an appropriate scale.

### 2.3. Microarray Analysis

Analysis of Variance (ANOVA) was performed to identify genes with differing means between more than two groups of conditions [36]. Partek (Missouri, USA) was used to incorporate contrasts within ANOVA, prioritizing the comparison of LPCs to other cell types. Genes with *p* values less than 0.01 were considered differentially expressed.

Principal component analysis (PCA) [37] was performed using Partek to visualize the natural grouping of the arrays based on global gene expression data. The three principal components that captured most differences were displayed in three-dimensional scatter plots. Similar microarrays are grouped together and more diverse microarrays are spaced further apart.

Expression datasets were hierarchically clustered in Partek under the parameters of complete (>1000 rows) or average (<1000 rows) linkage, Euclidean distance, and agglomerative clustering. Unsupervised clustering enabled relationships between rows and columns to be organized into tree diagrams without imposing subjective bias on the number or size of the clusters. Clusters were systematically colored and selected based on the distances calculated and displayed in the tree diagram branch arms using the “color by cluster” function in Partek.

The coefficient of variation was determined for each gene by dividing the standard deviation of each gene expression pattern by its average expression value, as previously described [38], to identify gene expression profiles with minimal variation across the cell types analyzed. The coefficient of variations and Pearson correlation coefficients were calculated in Microsoft Excel.

### 2.4. Pathway and Promoter Analysis

Database for Annotation, Visualization and Integrated Discovery (DAVID) was used to ascertain overrepresented pathways and perform gene identifier conversions. The pathway overrepresentation used an EASE score (a modified Fisher exact test) to calculate *p* values and determine if proportions of the categories differed [39, 40]. A significance threshold was set to *p* < 0.05. Only pathways that met a *p* < 0.05 threshold and contained 10 or more genes from the input cluster were included in our analysis.

Promoter Analysis and Interaction Network Toolset (PAINT) was used to search for overrepresented transcription factor binding elements in gene promoters [41]. The TransFac public database containing known transcription factor binding sites was used to search 2000 bases upstream of the transcriptional start sites of the input Entrez Gene lists. Other parameters selected were the match filter option set to minimize false positives, the core similarity threshold set to 1.0, and binding elements searched on both strands of DNA.

### 2.5. qPCR Analysis to Confirm Presence of Predicted LPC Markers in Liver Tissue

Mice were subjected to one of four regimes: 3,5-diethoxy-carbonyl-1,4-dihydrocollidine (DDC, *n* = 4) induced hepatotoxicity, CDE injury (*n* = 6), or one of two transgenic models of immune-mediated hepatitis, Met-Kb (*n* = 3) and 178.3 (*n* = 3). Experimental mice were subjected to the DDC regime for 28 days or 21 days for the remaining models. Controls (*n* = 3, 8, 3, 3, resp.) were used to contrast gene expression levels. All animal experiments complied with the guidelines specified by the National Health and Medical Research Council of Australia. Total RNA was extracted from the livers of the mice described above and cDNA was transcribed using Tetro RT*™* reverse transcriptase (Bioline). TaqMan two-step, real-time quantitative PCR (qPCR) with hydrolysis probes from the Universal Probe Library and the Light Cycler 480 (LC480) Probe Mastermix (Roche) was used to quantify mRNA expression. Genes selected to predict the presence of LPCs in injured livers were muscle-restricted coiled-coil protein (*Murc*) from group *α*, latent transforming growth factor beta binding protein 3 (*Ltbp3*) and neural cell adhesion molecule 1 (*Ncam1*) from group *β*, and minichromosome maintenance deficient (*Mcm2*) and pyruvate kinase isozyme M2 (*M2pk*) from group *γ*. The expressions of known LPC markers,* Cd24a* antigen and* Sox9*, were also determined. Primer sequences are available in Supplementary Table 2. Data was analyzed using the LC480 Relative Quantification Software. A five-point standard curve was generated for each gene, using pooled cDNA of each sample. A calibrator sample was also included in every qPCR experiment, for which each qPCR run was replicated three times. Data was included when amplification efficiencies/correlation for the assay was ≥95%. Significant changes in gene expression were determined by comparing control to experimental samples using a two-tailed Student's *t*-test. Data are displayed relative to TATA-box associated factor 4A (*Taf4a*) mRNA expression and normalized to controls.

### 2.6. Quantifying Liver Progenitor Cell Numbers* In Vivo*

Sections of formalin-fixed, paraffin-embedded CDE, DDC, Met-Kb, and 178.3 mouse liver were dewaxed and stained with hematoxylin and eosin to ascertain liver morphology or were stained with anti-panCK to determine LPC response. PanCK staining was achieved by first performing antigen retrieval using proteinase K (Dako, VIC) before applying a 1 : 400 dilution of anti-panCK (#Z0622, Dako) overnight at 4°C. Staining was processed and visualized with DAB using the LSAB system (Dako) according to the manufacturer's instructions. Stained sections were scanned at 40x magnification using an Aperio ScanScope XT. The Positive Pixel Count v9.1 algorithm within the ImageScope software was used to determine LPC response a.k.a. “panCK positivity,” the number of pixels positive for panCK staining as a percentage of total tissue pixels.

## 3. Results

### 3.1. Principal Component Analysis Confirms Successful Normalization of Microarrays

Since many of the microarray experiments were performed in different laboratories, nonbiological variables resulting in differing gene expression and transcript abundance are expected. These factors include chip-to-chip variability, different culture conditions including medium additives (e.g., growth factors and serum), cell density at time of harvest, RNA quality/quantity, and differences in reagents, kits, and equipment. It was therefore necessary to normalize the data before comparisons were made. All raw microarray data were subject to identical linear scaling and normalization to reduce the impact of nonbiological factors.

**Figure 1:**
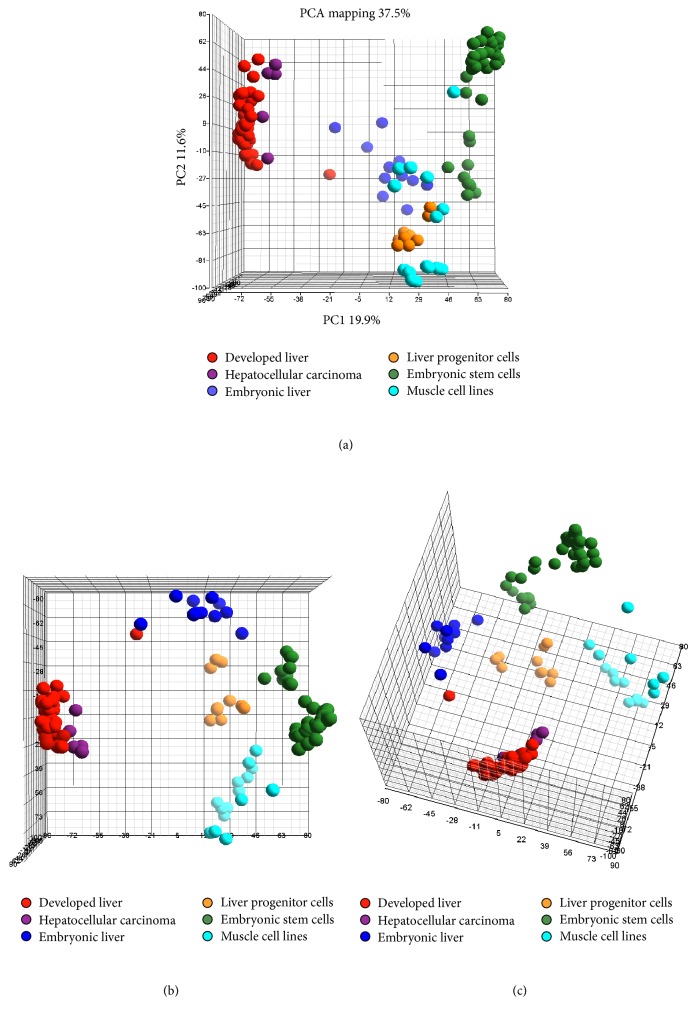
Principal component analysis (PCA) displays transcriptome clustering sorted by tissue type. Each node represents the average of at least 3 microarray replicates and is color-coded according to tissue type. The three principal components that captured most differences in the datasets are plotted with three different three-dimensional views of the same PCA visualization shown in (a), (b), and (c).

To test the success of this normalization, PCA was performed and the three principal components that captured most differences (37.5% of the total variation) in the datasets were plotted (Figure 1). PC1, PC2, and PC3 captured 19.9%, 11.6%, and 6.0% of variation in the datasets, respectively, and are shown from three angles to aid in distinguishing each cell/tissue type cluster (Figures 1(a)–1(c)). Without adequate normalization, different arrays would likely appear scattered across the three-dimensional PCA plot. In this instance, the normalized arrays clearly grouped on the PCA plot according to tissue type, and differences between tissue types were greater than those within each grouping. For instance, LPC lines (orange nodes) clustered closely together and away from DL microarrays (red nodes). Of all cell/tissue groupings, the DL and HCC arrays clustered closest together, yet they still occupied distinct regions. This distinction is best visualized on the first principal component (PC1), which accounts for the greatest differences in the analysis (Figure 1(a)). Crucially all data was generated in an unsupervised manner, that is, without introducing bias from tissue type. These findings were demonstrative of successful normalization and justified subsequent analyses to identify a distinct LPC gene expression pattern.

### 3.2. Analysis of Hierarchical Clusters Reveals Distinct LPC Expression Profiles, Pathways, and Promoters

**Figure 2:**
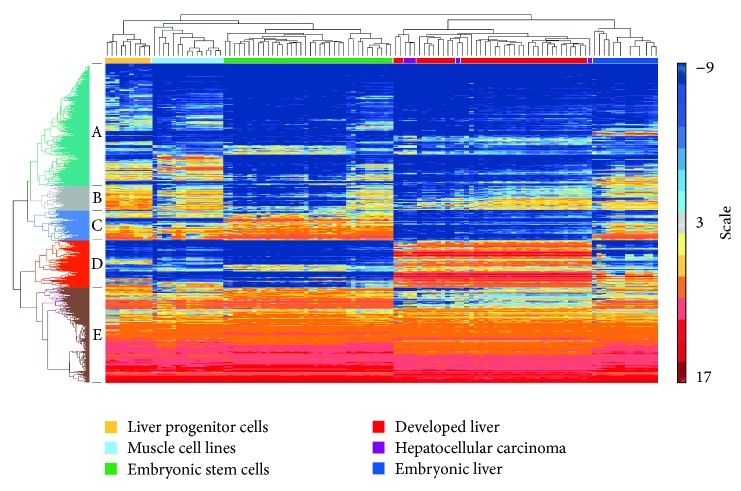
Hierarchical clustering identifies 5 clusters (A, B, C, D, and E) of distinct gene expression data for liver progenitor cells. ANOVA was performed to compare liver progenitor cells to all other cell/tissue type groups. The 8,623 probe sets that displayed expression levels significantly different to LPCs (*p* < 0.01) were subsequently clustered according to the parameters of Euclidean distance and complete linkage.

ANOVA was applied to the tissue type groupings to distinguish the LPC transcriptome from other cell types. Analysis identified 8623 differentially expressed probe sets when comparing LPCs to all other groups. From this dataset, five unique clusters were identified and named clusters A to E (Figure 2). Cluster A represents probe sets with equivalent or higher expression in LPCs compared to other cell types. Cluster B includes probes that display high variability within similar tissue types and thus is largely uninformative. Cluster C represents probe sets with high expression in “immortalized” cell lines including LPCs, muscle, embryonic stem cells, and embryonic liver whilst being poorly expressed in DL and HCC. Cluster D includes probes with higher expression in DL and HCC samples, and cluster E represents probe sets whose expressions were highly abundant in the majority of samples. Clusters A, C, and D presented the greatest differences between LPCs and other cell/tissue groups and thus were the focus of further analysis. Figure 2 data is provided as a supplementary spreadsheet (Figure 2 Matrix).

**Figure 3:**
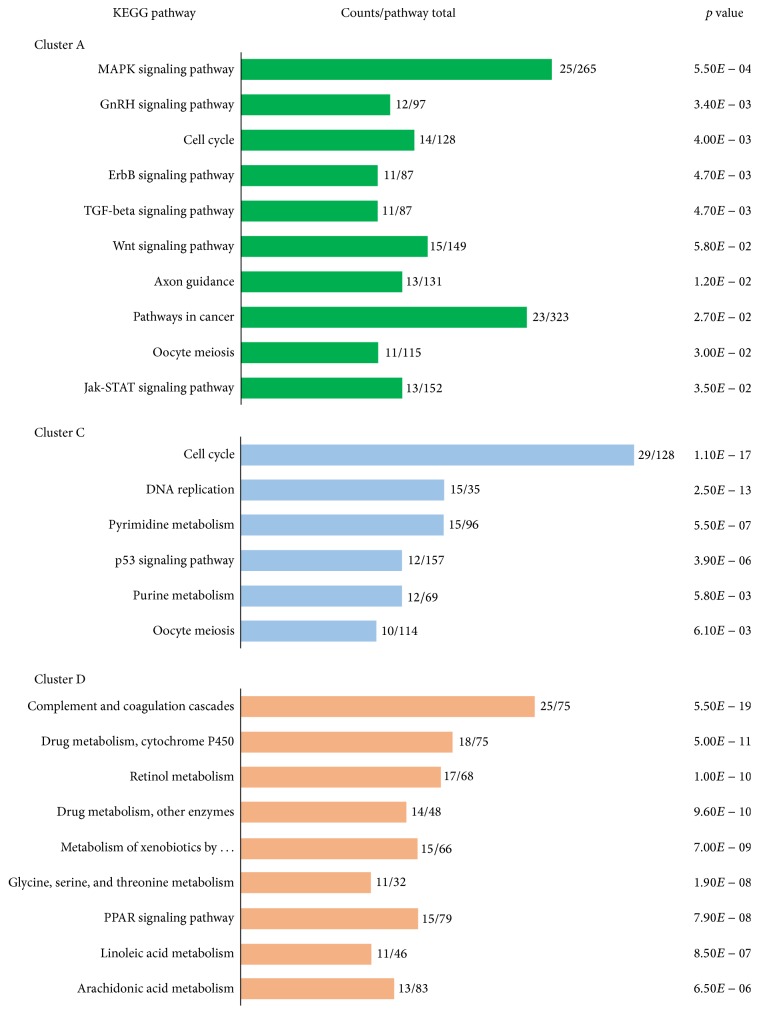
Probe set clusters A, C, and D contain pathways that are overrepresented in LPCs and liver tissue. Clusters A and C contain probe sets that are upregulated in liver progenitor cells (LPCs) and cluster D contains those that are upregulated in developed liver whilst being low in LPCs. The Database for Annotation, Visualization and Integrated Discovery online tool was used to identify overrepresented pathways (*p* < 0.05) within each of these clusters. Pathways are displayed together with the number of genes from the cluster list (counts), the total that belongs to each pathway (pathway total) and corresponding *p* values.

As evident from the scattered color within each cluster, not all probe sets closely aligned within each cluster. Stringent filters were applied to more precisely define the LPC transcriptome. The MAS 5.0 algorithm was applied to make present/absent calls for probe sets. For clusters A and C, only probe sets that were present in 90% of LPC arrays were retained. In contrast, only sets absent in 90% of LPC arrays were retained for cluster D, since they were largely absent in LPCs. The DAVID and KEGG pathway tools were subsequently applied to the truncated cluster A, C, and D lists to functionally categorize and identify patterns of biological significance within each. The resulting overrepresented pathways (*p* < 0.05), the number of cluster genes found (counts), and the total genes within each pathway (pathway total) are shown in Figure 3.

DAVID was able to annotate 254 genes to KEGG pathways from the filtered cluster A probe set list. In the overrepresented mitogen-activated protein kinase (MAPK) signaling pathway there were 25 counts from a MAPK pathway total. Other cluster A pathways included GnRH signaling, cell cycle, transforming growth factor- (TGF-) beta signaling, and epidermal growth factor (EGF) receptor ErbB signaling. Within this cluster, PAINT analysis identified nuclear factor (erythroid-derived 2)-like 2 (*Nrf2*), upstream stimulatory factor (*Usf1*), cyclic-AMP response element binding protein (*Creb*), and activating transcription factor (*Atf1*) as predicted binding elements playing an important role in the coregulation of LPC associated genes.

Using the truncated cluster C probe set list, DAVID annotated 426 genes to KEGG pathways. Overrepresented pathways included cell cycle, DNA replication, pyrimidine and purine metabolism, and p53 signaling pathways (Figure 3). There were also several overrepresented predicted binding elements for cluster C including growth factor independent 1 transcription repressor (*Gfi1*), E2F transcription factor 1 (*E2f1*), melanocyte-inhibiting factor (*Mif*), and cartilage oligomeric matrix protein (*Comp*).

Clusters A and C are both highly expressed in LPCs whereas probe sets in cluster D are lower in LPCs compared to DL and HCC samples (Figure 2). The 90% absent filtered list for cluster D was annotated to 359 genes and overrepresented KEGG pathways were involved in fundamental liver functions, including immunity, detoxification, and lipid metabolism. Some of these pathways were complement and coagulation cascades, peroxisome proliferator-activated receptor (PPAR) signaling, linoleic acid, and multiple metabolic pathways. Following PAINT analysis, overrepresented promoter elements discovered include jun oncogene, hepatocyte nuclear factors 1 and 4 (*Hnf1* and* Hnf4*), and nuclear factor of kappa light polypeptide gene enhancer in B cells (*NfκB*), many of which support liver function.

The probe set IDs (ordered according to pathway), gene names, symbols, and the fold change between LPC and DL for clusters A, C, and D are displayed in Supplementary Tables 3–5. A summary of the promoter elements for these clusters is available as Supplementary Table 6.

### 3.3. The LPC Transcriptomic Profile Reveals Known and Novel Marker Genes

To identify individual genes representative of LPCs, probe sets corresponding to known or widely used LPC markers were selected. These included A6 actin-binding protein, alpha fetoprotein, albumin, CD34 antigen, keratins 7, 8, 14, 18, and 19, c-kit oncogene, Cx32 and Cx43 gap junction proteins, Delta-like 1 homolog, gamma glutamyl transferase 1, pyruvate kinase isoenzyme type M2, glutathione S-transferase, ataxin 1, epithelial cadherin, and neural cell adhesion molecule [25, 42–45]. Pearson correlation coefficients were calculated for comparisons between the above probe sets and each of those in Figure 2. This provided a statistical relationship between the known LPC markers and the total list of differentially expressed probe sets in LPCs and produced a list of 355 sets with a correlation >0.9.

**Figure 4:**
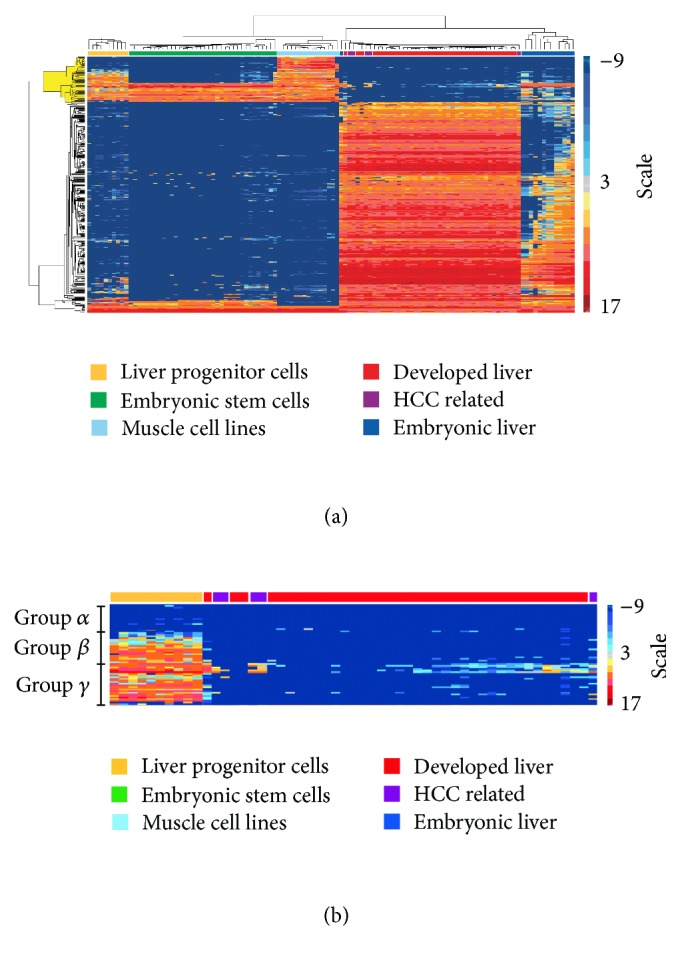
Distinct expression profiles for liver progenitor cells identify three gene expression groups. (a) Hierarchical clustering of expression profiles that correlate with known liver progenitor cell markers. (b) Close view of the yellow highlighted rows from (a), with particular focus on the LPC and developed liver groupings. Three distinct groups of gene expression patterns (*α*, *β*, and *γ*) are defined.

These probe sets were hierarchically clustered to visualize expression patterns in various tissue types (Figure 4(a)). Figure 4(a) data is provided as a supplementary spreadsheet (Figure 4A Matrix). Although all probe sets correlated with LPC markers, not all are highly expressed in LPCs. This can be explained as LPC markers include a mixture of hepatocyte and cholangiocyte markers, which may not be expressed by all types of LPCs.  Figure 4(b) is a magnification of an area of interest highlighted in yellow in Figure 4(a). We focused on LPC and DL tissues since diagnostically it is most beneficial for LPCs to be genetically distinguished from DL. As expected from the PCA, HCC samples are interspersed within the DL arrays (Figure 4(b)). Interestingly LPCs share some common expression patterns with HCC samples, perhaps indicative of their tumorigenic potential. To further investigate this, probe sets with low expression in DL but at least two-fold greater expression in HCC and LPC categories were identified. These were then compared to genes displaying a similar expression pattern within hepatocytes, fibrolamellar HCCs, and hepatoblasts by Oikawa et al. [27] (Supplementary Table 7). We designated the distinct clustered groupings in Figure 4(b): *α*, *β*, and *γ*. Group *α* probe sets have low expression in LPCs, whilst those in groups *β* and *γ* are highly expressed.

Lastly, to generate a list of LPC-expressed genes, the 355 probe sets of Figure 4(a) were refined by retaining only those that were present in at least 9 of the 10 averaged LPC microarrays. This filtering yielded 40 probe sets annotated to 34 unique genes, shown in Table 1 including 8 genes that have been previously associated with LPCs.

### 3.4. Identified Markers Detect LPCs in Injured Liver Tissue and Their Expression Levels Correlate with LPC Numbers

For detection of LPCs within a liver sample, genes from group *α* should display low expression, whilst genes from groups *β* and *γ* should exhibit high expression, relative to a reference gene. An ideal reference gene should have little to no variability across a variety of tissue types, and thus it should have a low coefficient of variation.* Taf4a* on the Affymetrix Mouse Genome 430 2.0 chip fits this criterion.

**Figure 5:**
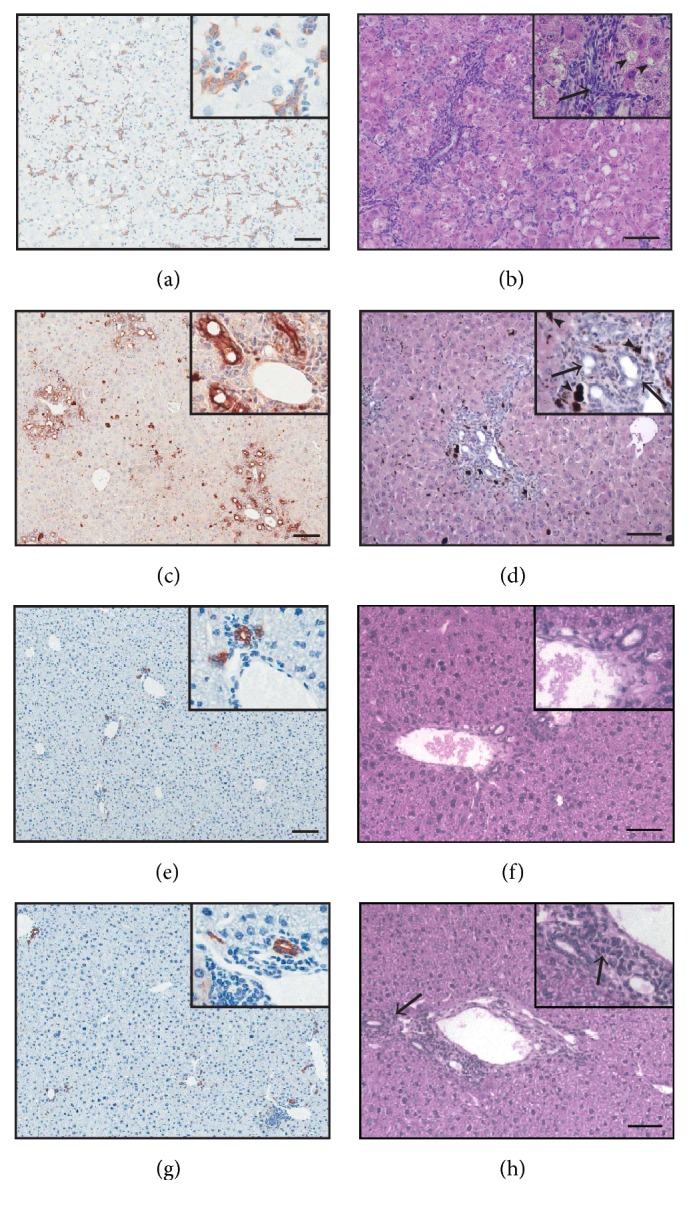
Four liver injury models display different degrees of pathology and liver progenitor cell (LPC) response. Liver sections from wild-type mice subjected to a choline-deficient, ethionine-supplemented (CDE; (a) and (b)) or 3,5-diethoxy-carbonyl-1,4-dihydrocollidine (DDC; (c) and (d)) diet, or transgenic 178.3 ((e) and (f)) or Met-Kb ((g) and (h)) mice were stained with panCK ((a), (c), (e), and (g)) or H&E ((b), (d), (f), and (h)). Arrows in panels (b) and (d) and (h) indicate LPCs, ductular reactions, and small basophilic cells, respectively. Arrowheads in panels (b) and (d) indicate steatosis and porphyrin accumulations, respectively. Scale bars represent 100 *μ*m.

To determine whether a combination of selected genes from groups *α*, *β*, and *γ* could accurately detect the presence and abundance of LPCs in liver tissue of varying pathologies we obtained the livers of mice subjected to the CDE diet, the immune-mediated Met-Kb and 178.3 transgenic mouse models, and DDC induced hepatotoxicity. The histology of these livers showed varying degrees of disease severity (Figure 5). CDE liver samples displayed steatosis (Figure 5(b), arrowheads) and numerous small basophilic cells with oval-shaped nuclei (Figure 5(b), arrows) indicative of LPCs. The DDC liver featured porphyrin accumulations (Figure 5(d), arrowheads) and ductular reactions (Figure 5(d), arrows), whilst the 178.3 liver (Figure 5(f)) displayed normal architecture and Met-Kb liver showed increased ductal structures surrounded by many small basophilic cells (Figure 5(h), arrows). The degree of LPC induction was determined by staining and quantification of panCK positivity (Figures 5(a), 5(c), 5(e), and 5(g)), found to be 3.3%, 2.5%, 0.09%, and 0.11% for CDE, DDC, 178.3, and Met-Kb models, respectively.

**Figure 6:**
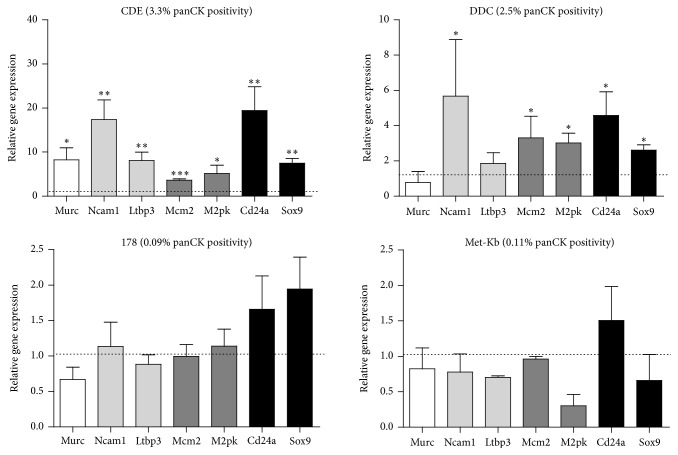
A liver progenitor cell (LPC) transcriptomic signature can identify liver injury models with LPC induction. qPCR-generated mRNA expression levels of known (*Cd24a* and* Sox9*) and identified (group *α*,* Murc*; *β*,* Ncam1* and* Ltbp3*; and *γ*,* Mcm2* and* M2pk*) LPC markers in four different liver injury models. Data are normalized to gene expression in the appropriate control livers (indicated by the dotted line) and are relative to the* Taf4a* housekeeping gene. Data represents mean + SEM of 3 separate qPCR assays with significance determined by Student's *t*-test (^*∗*^ *p* < 0.05, ^*∗∗*^ *p* < 0.01, and ^*∗∗∗*^ *p* < 0.001).

From our LPC gene signature* Ltbp3* and* Ncam1* from group *β* and* Mcm2* and* M2pk* from group *γ* displayed the highest abundance in livers of mice on diets yielding the greatest proportion of panCK positivity (Figure 6). Protein expression of NCAM1 isoform 140 and M2PK was increased in 75% CDE diet livers relative to controls, as determined by Western blot (Supplementary Figure 1). No clear pattern was observed within NCAM1 isoform 180. The group *α* gene* Murc* that is expressed at low levels in LPCs and DL showed decreased expression in DDC, Met-Kb, and 178.3 liver but surprisingly was increased in CDE liver (Figure 6).

**Figure 7:**
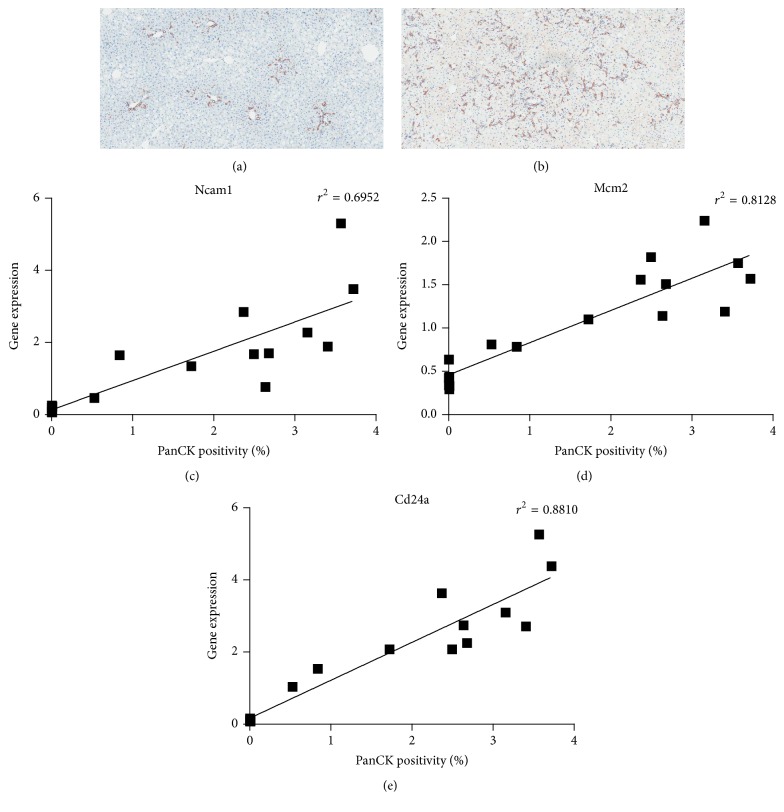
Gene expression of group *β* and *γ* genes,* Ncam1* and* Mcm2*, and the known marker* Cd24a* correlate with panCK staining* in vivo*. Representative images of (a) control and (b) choline-deficient, ethionine-supplemented (CDE) mouse liver sections stained for panCK. Expressions of (c)* Ncam1*, (d)* Mcm2*, and (e)* Cd24a* relative to* Taf4a* in a variety of CDE and control liver tissues are plotted against their corresponding panCK positivity values.

To confirm that transcript abundance of genes in groups *β* and *γ* correlates with LPC number, we quantified the LPC response in day 0 (*n* = 7) and day 21 (*n* = 11) mouse CDE livers (Figure 7). LPC quantitation for all samples ranged between 0.0036% (Figure 7(a)) and 3.7% (Figure 7(b)). Predictably, mRNA levels for an established LPC marker* Cd24a* strongly correlated (*r* ^2^ = 0.8810) with panCK positivity (Figure 7(e)). Importantly, expression of* Ncam1* (group *β*) and* Mcm2* (group *γ*) also correlated well (*r* ^2^ = 0.6952 and 0.8128, resp.) with panCK staining (Figures 7(c) and 7(d)). Collectively these results validate our approach to identify novel LPC markers that can be used for their detection* in vivo* and their utility to assess liver pathology with respect to the extent of LPC induction.

## 4. Discussion

In this study we demonstrate that it is possible to collect, analyze, and compare publicly available transcriptome data to derive useful information relating to a particular cell type of interest, in this instance, LPCs. This is possible so long as appropriate criteria for their inclusion are adopted, and it is important to use related microarray platforms with a minimum of three highly correlated replicates and consistent data normalization. Adherence to these principles benefited this study as it integrated more than 400 microarrays to highlight a unique transcriptomic signature of LPCs.

PCA successfully separated LPCs from other stem cell types and more importantly from other liver cell and tissue samples. Notably, LPC lines from different research groups, including array data from a BMEL line derived in Paris and analyzed in Houston [46], T-LPC isolated in Tokyo [22] and analyzed in Perth, and other lines isolated and analyzed in Perth, were more similar to each other than to other tissue or cell types. Spatially, the LPC cluster is distant from DL, not surprising considering their different functions and degree of differentiation. Given the association between LPCs and HCC, closer clustering of these two groups could be expected. However, the LPC and HCC clusters are distant, indicating these HCCs may not be derived from LPCs. Indeed one of the five HCC analyses used in this study mentions that the tumors are mostly well-differentiated [47]. In this case we would expect the HCC group to cluster closer to the DL group than the LPC one, as reported in this study. Unfortunately, there is limited or no information available regarding the differentiated status of the remaining four HCCs used [48, 49]. Histologically, poorly differentiated HCCs often carry LPC and/or stem markers [50, 51] and may be more indicative of LPC-derived HCC. Use of these types of tumors may have offered further insights into the relationship between LPCs and LPC-derived HCC. Moreover LPCs cluster closely with embryonic liver due to their shared plasticity and origin. In fact the BMEL LPC line used was derived from embryonic liver. The proximity of LPCs to muscle is unexpected, though it may be a result of their shared immortalized progenitor cell status. LPCs and C2C12 myoblasts used in this study represent immature cells of their respective lineages and are both involved with tissue regeneration. Comparison of primary LPCs and muscle cells may have prevented this association. Despite this, our use of immortalized cell lines yielded a transcriptomic signature consistent with LPC biology and genetics.

Notably, overrepresented pathways in cluster D, which were poorly expressed in LPCs but highly expressed in developed liver, were consistent with liver functions including drug, vitamin, and fatty acid metabolism. Furthermore upregulated LPC pathways in clusters A and C are consistent with transcriptomic analyses of rat oval cells [52]. These LPC pathways included the Wnt, ErbB, p53, MAPK, and TGF-beta signaling pathways. Wnt/*β*-catenin is a mediator of LPC activation and expansion [53]. Wnt induced *β*-catenin phosphorylation targets the gene promoters of EGF, cyclin D (*Ccnd1*), fos-like antigen 1 (*Fosl1*), and c-Myc, to drive cellular proliferation [54, 55]. Consistently, in cluster A* Ccnd1* and* Fosl1* were upregulated in LPCs. Furthermore, activation of cancer-related processes through* Myc* and* Ccnd1* and matrix metalloproteinases have been identified [56] and increased *β*-catenin expression has been reported in HCC [57] and aggressive hepatoblastomas [58].

The ErbB signaling pathway affects diverse cellular functions including proliferation, angiogenesis, migration, and differentiation. An* ErbB2* overexpressing transgenic mouse exhibits increased numbers of LPC-like cells in the liver [59], and increased expression of ErbB family members is reported in a high proportion of human HCCs and correlates with cancer progression [60]. These findings support the integral role of the ErbB family in liver carcinogenesis. It is widely accepted that aberrant p53 signaling participates in the development of HCC [61]. Moreover the MAPK signaling pathway is associated with tumorigenesis via regulation of proliferation, differentiation, survival, and migration [62]. Finally, increased TGF-beta signaling is critical in inhibition of hepatocyte-mediated regeneration [63], promoting LPC-mediated regeneration instead [64]. These results highlight LPCs as a regenerative cell source for the liver but also as a putative cancer stem cell that may give rise to HCC [53, 65].

The individual LPC genes identified in this study (Table 1) also reflect LPC function.* Mcm2* is expressed in a variety of regenerative cells including neural stem cells and hepatocytes [66, 67].* Fhl2*,* Klf5*, and* Enah* are highly expressed in rat embryonic hepatoblasts relative to adult hepatocytes [68] and* Fhl2* is a marker of progenitor cells of myeloid [69], myocardial [70], and mesenchymal [71] origin.* Ndrg4* is a gene involved in cell cycle progression and survival [72].* Abcc1* is highly expressed in stem-like cells within HCC cell lines and confers chemotherapeutic resistance, a property of HCC [73].

In this study we were able to use genes from our LPC signature to successfully reflect the presence or absence of LPCs in a number of liver injury models. The unexpected expression of the group *α* gene,* Murc*, in CDE liver may be explained by the presence of activated hepatic stellates in this model [74], since they are known to express* Murc* [75]. Consequently, we found expression of individual genes from either group *β* or group *γ* was more reliable for detection of LPCs in tissue samples than a signature encompassing all three groups.

Our approach has identified many novel LPC markers, useful for detection of these cells* in vivo*. Markers that are expressed on the cell surface have the potential for isolating LPCs. According to their gene ontology, the novel LPC markers GNBP5, PANX1, PRKG2, and TULP3 from Table 1 localize to the plasma membrane; however this would need to be confirmed using fluorescence microscopy and subcellular fractionation.

## 5. Conclusion

Using publicly available gene expression/microarray data, we compare the LPC transcriptome against other cell and tissue types and identify signaling pathways that support their ability to rapidly proliferate and differentiate. We find pathways which are consistent with their putative role as a cancer stem cell in HCC. Importantly we also identify many novel LPC markers, several of which localize to the cell membrane. This is beneficial for the field as there is a scarcity of specific markers that can both identify and purify LPCs. Finally, since LPCs may reflect a precancerous liver condition as well as the severity for a range of liver pathologies, our LPC signature could ideally be used as a novel early indicator of liver disease in patients.

## Supplementary Material

The supplementary materials contain; a figure pertaining to M2PK and NCAM1 protein expression, spreadsheets corresponding with Figure 2 and 4a, a list of all microarrays used in this meta-analysis and their source, primer sequences, genes corresponding to the overrepresented pathways identified for clusters A, C and D, a summary of the promoter analysis data and a comparison between our dataset and one generated by Oikawa T, et al. in an investigation of Fibrolamellar hepatocellular carcinoma.

## Figures and Tables

**Table 1:** Markers for liver progenitor cells (LPCs): expression profiles that correlate closely with known LPC markers were mined for potential marker genes. Gene symbol, title, and transcript abundance fold change in LPCs relative to developed liver (DL). References to studies that identified genes as LPC markers are included where applicable.

Gene symbol	Gene title	Fold change (LPC/DL)	References

Abcc1	ATP-binding cassette, subfamily C (CFTR/MRP) 1	152	[73]
Aldh18a1	Aldehyde dehydrogenase 18 family, member A1	66.1	
Capg	Capping protein (actin filament), gelsolin-like	82.6	
Cdc20	Cell division cycle 20 homolog (*S. cerevisiae*)	16.5	
Enah	Enabled homolog (*Drosophila*)	Not expressed in DL	[68, 76]
Fhl2	Four and a half LIM domains 2	Not expressed in DL	[68]
Gdpd1	Glycerophosphodiester phosphodiesterase domain 1	140	
Gnb5	Guanine nucleotide binding protein (G protein), beta 5	92.3	
Gpr177	G protein-coupled receptor 177	107	
Hs2st1	Heparan sulfate 2-O-sulfotransferase 1	Not expressed in DL	
Ift57	Intraflagellar transport 57 homolog (*Chlamydomonas*)	29.8	
Klf5	Kruppel-like factor 5	506	[68]
Ltbp3	Latent transforming growth factor beta binding protein 3	Not expressed in DL	
Luzp1	Leucine zipper protein 1	77.7	
Map4k4	Mitogen-activated protein kinase kinase kinase kinase 4	34.2	[77]
Mcm2	Minichromosome maintenance deficient 2	8.53	
Ncam1	Neural cell adhesion molecule 1	Not expressed in DL	[78]
Ndrg4	N-myc downstream regulated gene 4	146	
Npnt	Nephronectin	83.4	
Nup93	Nucleoporin 93	10.3	
Nxn	Nucleoredoxin	16.9	[76]
Panx1	Pannexin 1	Not expressed in DL	
Pfkp	Phosphofructokinase, platelet	Not expressed in DL	
Phc1	Polyhomeotic-like 1 (*Drosophila*)	Not expressed in DL	
Pkia	Protein kinase inhibitor, alpha	Not expressed in DL	
Pkm2	Pyruvate kinase, muscle	115	[79]
Plod2	Procollagen lysine, 2-oxoglutarate 5-dioxygenase 2	Not expressed in DL	
Pmm1	Phosphomannomutase 1	68.2	
Prkg2	Protein kinase, cGMP-dependent, type II	Not expressed in DL	
Sfxn3	Sideroflexin 3	92.5	
Tnnt2	Troponin T2, cardiac	Not expressed in DL	
Tubb2b	Tubulin, beta 2b	818	
Tulp3	Tubby-like protein 3	Not expressed in DL	
Wisp1	WNT1 inducible signaling pathway protein 1	69.0	

## Abbreviations

BMEL: Bipotential murine embryonic liver
BMOL: Bipotential murine oval liver
CDE: Choline-deficient, ethionine-supplemented
DAVID: Database for Annotation, Visualization and Integrated Discovery
DDC: 3,5-Diethoxy-carbonyl-1,4-dihydrocollidine
DL: Developed liver
GEO: Gene Expression Omnibus
HCC: Hepatocellular carcinoma
KEGG: Kyoto Encyclopedia of Genes and Genomes
LPC: Liver progenitor cell
MAD: Median Absolute Deviation
MAS 5.0: MicroArray Suite 5.0
PAINT: Promoter Analysis and Interaction Network Toolset
PCA: Principal component analysis
PIL: p53-immortalized liver
T-LPC: Tokyo-LPC.
