# Supplementary material for: A Transcriptomic Signature of Mouse Liver Progenitor Cells
Source: Stem Cells Int. 2016 Oct 3;2016:5702873. doi: 10.1155/2016/5702873 (PMC5061959; doi:10.1155/2016/5702873)
Supplement: Supplementary file 1 — The supplementary materials contain; a figure pertaining to M2PK and NCAM1 protein expression, spreadsheets corresponding with Figure 2 and 4a, a list of all microarrays used in this meta-analysis and their source, primer sequences, genes corresponding to the overrepresented pathways identified for clusters A, C and D, a summary of the promoter analysis data and a comparison between our dataset and one generated by Oikawa T, et al. in an investigation of Fibrolamellar hepatocellular carcinoma. [file 5702873.f1.zip › Supplementary Figures.docx]

**
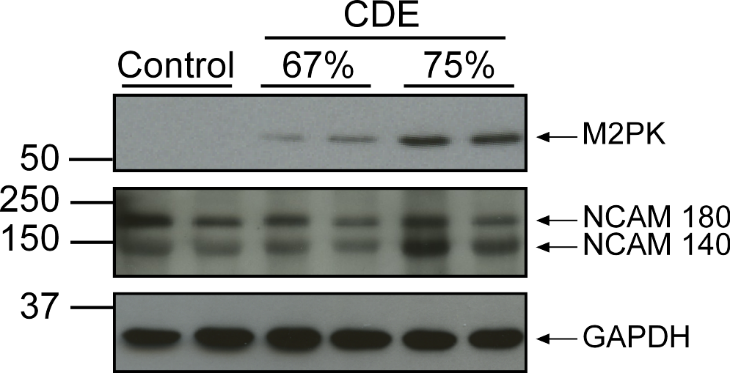
**

**Supplementary Figure S1**

Livers from mice fed a Control diet, a 67% or a 75% choline-deficient, ethionine-supplemented (CDE) diet were lysed and 60 µg of lysate was separated by SDS-PAGE. Proteins were transferred to a nitrocellulose membrane and immunoblotted for M2PK, NCAM1 and the loading control GAPDH. Size markers are shown in kilodaltons.
