# Supplementary material for: A Transcriptomic Signature of Mouse Liver Progenitor Cells
Source: Stem Cells Int. 2016 Oct 3;2016:5702873. doi: 10.1155/2016/5702873 (PMC5061959; doi:10.1155/2016/5702873)
Supplement: Supplementary file 1 — The supplementary materials contain; a figure pertaining to M2PK and NCAM1 protein expression, spreadsheets corresponding with Figure 2 and 4a, a list of all microarrays used in this meta-analysis and their source, primer sequences, genes corresponding to the overrepresented pathways identified for clusters A, C and D, a summary of the promoter analysis data and a comparison between our dataset and one generated by Oikawa T, et al. in an investigation of Fibrolamellar hepatocellular carcinoma. [file 5702873.f1.zip › Supplementary Tables FINAL.docx]

**Supplementary Table 1.** A summary of information pertaining to the 405 individual microarrays analysed. The ref. series “GSE85114” contains the arrays performed within this laboratory.

| **Tissue** | ***Array name*** | **Array ID** | **Platform** | **Experiment conditions** | **Ref Series** | **Notes** |
| --- | --- | --- | --- | --- | --- | --- |
| **Liver Progenitor Cells** | *LPC1R1* | GSM2257924 | 430 2.0 | PIL1 p40 | GSE85114 |  |
|  | *LPC1R2* | GSM2257925 |  | PIL1 p40 |  |  |
|  | *LPC1R3* | GSM2257926 |  | PIL1 p40 |  |  |
|  | *LPC2R1* | GSM2257927 | 430 2.0 | PIL2 p25 |  |  |
|  | *LPC2R2* | GSM2257928 |  | PIL2 p25 |  |  |
|  | *LPC2R3* | GSM2257929 |  | PIL2 p25 |  |  |
|  | *LPC2R4* | GSM2257930 |  | PIL2 p17 |  |  |
|  | *LPC3R1* | GSM2257931 | 430 2.0 | PIL3 p32 |  |  |
|  | *LPC3R2* | GSM2257932 |  | PIL3 p32 |  |  |
|  | *LPC3R3* | GSM2257933 |  | PIL3 p32 |  |  |
|  | *LPC4R1* | GSM2257934 | 430 2.0 | PIL4 p23 |  |  |
|  | *LPC4R2* | GSM2257935 |  | PIL4 p23 |  |  |
|  | *LPC4R3* | GSM2257936 |  | PIL4 p23 |  |  |
|  | *LPC4R4* | GSM2257937 |  | PIL4 p15 |  |  |
|  | *LPC5R1* | GSM2257938 | 430 2.0 | PIL5 p40 |  |  |
|  | *LPC5R2* | GSM2257939 |  | PIL5 p40 |  |  |
|  | *LPC5R3* | GSM2257940 |  | PIL5 p40 |  |  |
|  | *LPC6R1* | GSM2257941 | 430 2.0 | BMOL p25 |  |  |
|  | *LPC6R2* | GSM2257942 |  | BMOL p25 |  |  |
|  | *LPC6R3* | GSM2257943 |  | BMOL p25 |  |  |
|  | *LPC6R4* | GSM2257944 |  | BMOL p19 |  |  |
|  | *LPC7R1* | GSM2257945 | 430 2.0 | T-LPC p49 |  |  |
|  | *LPC7R2* | GSM2257946 |  | T-LPC p49 |  |  |
|  | *LPC7R3* | GSM2257947 |  | T-LPC p49 |  |  |
|  | *LPC8R1* | GSM160432 | 430A | BMEL 9A1 | GSE6942 |  |
|  | *LPC8R2* | GSM160433 |  | BMEL 9A1 |  |  |
|  | *LPC8R3* | GSM160071 | 430 2.0 | BMEL 9A1 | GSE6957 |  |
|  | *LPC8R4* | GSM160072 |  | BMEL 9A1 |  |  |
|  | *LPC9R1* | GSM160073 | 430A | BMEL 14B3 | GSE6942 |  |
|  | *LPC9R2* | GSM160074 |  | BMEL 14B3 |  |  |
|  | *LPC9R3* | GSM160434 | 430 2.0 | BMEL 14B3 | GSE6957 |  |
|  | *LPC9R4* | GSM160435 |  | BMEL 14B3 |  |  |
|  | *LPC10R1* | GSM323977 | 430 2.0 | BMELs transfected with a control random oligo | GSE12908 |  |
|  | *LPC10R2* | GSM323978 |  |  |  |  |
|  | *LPC10R3* | GSM323979 |  |  |  |  |
|  | *LPC10R4* | GSM323980 |  |  |  |  |
|  | *LPC10R5* | GSM323981 |  |  |  |  |
| **Embryonic Liver** | *ELiv1R1* | GSM177034 | 430 2.0 | Liver from WT embryonic mice day 13.5 | GSE7342 |  |
|  | *ELiv1R2* | GSM177035 |  |  |  |  |
|  | *ELiv1R3* | GSM177036 |  |  |  |  |
|  | *Eliv2R1* | GSM177040 | 430 2.0 | Liver from WT embryonic mice day 15.5 |  |  |
|  | *Eliv2R2* | GSM177041 |  |  |  |  |
|  | *Eliv2R3* | GSM177042 |  |  |  |  |
|  | *ELiv3R1* | GSM161128 | 430 2.0 | Livers from WT mouse embryos 10.5 days post conception (from 3 pooled livers) | GSE6998 | Combined |
|  | *ELiv3R2* | GSM161129 |  |  |  |  |
|  | *ELiv3R3* | GSM161130 | 430 2.0 | Livers from WT mouse embryos 11.5 days post conception (from 3 pooled livers) |  |  |
|  | *ELiv3R4* | GSM161131 |  |  |  |  |
|  | *ELiv4R1* | GSM161132 | 430 2.0 | Livers from WT mouse embryos 12.5 days post conception (from 3 pooled livers) |  | Combined |
|  | *ELiv4R2* | GSM161133 |  |  |  |  |
|  | *ELiv4R3* | GSM161134 | 430 2.0 | Livers from WT mouse embryos 13.5 days post conception (from 3 pooled livers) |  |  |
|  | *ELiv4R4* | GSM161135 |  |  |  |  |
|  | *ELiv5R1* | GSM161136 | 430 2.0 | Livers from WT mouse embryos 14.5 days post conception (from 3 pooled livers) |  | Combined |
|  | *ELiv5R2* | GSM161137 |  |  |  |  |
|  | *ELiv5R3* | GSM161138 | 430 2.0 | Livers from WT mouse embryos 16.5 days post conception (from 3 pooled livers) |  |  |
|  | *ELiv5R4* | GSM161139 |  |  |  |  |
|  | *ELiv6R1* | GSM329271 | 430 2.0 | Fetal liver at E11.5 | GSE13149 | Combined |
|  | *ELiv6R2* | GSM329272 |  |  |  |  |
|  | *ELiv6R3* | GSM329273 | 430 2.0 | Fetal liver at E12.5 |  |  |
|  | *ELiv6R4* | GSM329274 |  |  |  |  |
|  | *ELiv7R1* | GSM329275 | 430 2.0 | Fetal liver at E13.5 |  | Combined |
|  | *ELiv7R2* | GSM329276 |  |  |  |  |
|  | *ELiv7R3* | GSM329277 | 430 2.0 | Fetal liver at E14.5 |  |  |
|  | *ELiv7R4* | GSM329278 |  |  |  |  |
|  | *ELiv8R1* | GSM329279 | 430 2.0 | Fetal liver at E15.5 |  | Combined |
|  | *ELiv8R2* | GSM329280 |  |  |  |  |
|  | *ELiv8R3* | GSM329281 | 430 2.0 | Fetal liver at E16.5 |  |  |
|  | *ELiv8R4* | GSM329282 |  |  |  |  |
|  | *ELiv9R1* | GSM329283 | 430 2.0 | Fetal liver at E17.5 |  | Combined |
|  | *ELiv9R2* | GSM329284 |  |  |  |  |
|  | *ELiv9R3* | GSM329285 | 430 2.0 | Fetal liver at E18.5 |  |  |
|  | *ELiv10R1* | GSM298115 | 430 2.0 | Fetal liver at E14.5 (Ter119+ cells) | GSE11777 |  |
|  | *ELiv10R2* | GSM298116 |  |  |  |  |
|  | *ELiv10R3* | GSM298117 |  |  |  |  |
|  | *ELiv11R1* | GSM282044 | 430 2.0 | CD-1 mouse liver 10.5 dpc | GSE11201 | Combined |
|  | *ELiv11R2* | GSM282045 |  |  |  |  |
|  | *ELiv11R3* | GSM282046 | 430 2.0 | CD-1 mouse liver 11.5 dpc |  |  |
|  | *ELiv11R4* | GSM282047 |  |  |  |  |
|  | *ELiv12R1* | GSM282048 | 430 2.0 | CD-1 mouse liver 12.5 dpc |  | Combined |
|  | *ELiv12R2* | GSM282049 |  |  |  |  |
|  | *ELiv12R3* | GSM282050 | 430 2.0 | CD-1 mouse liver 13.5 dpc |  |  |
|  | *ELiv12R4* | GSM282051 |  |  |  |  |
|  | *ELiv13R1* | GSM282052 | 430 2.0 | CD-1 mouse liver 14.5 dpc |  | Combined |
|  | *ELiv13R2* | GSM282053 |  |  |  |  |
|  | *ELiv13R3* | GSM282054 | 430 2.0 | CD-1 mouse liver 16.5 dpc |  |  |
|  | *ELiv13R4* | GSM282055 |  |  |  |  |
|  | *ELiv14R1* | GSM136904 | 430 2.0 | Livers from WT mice E14.5 | GSE5891 |  |
|  | *ELiv14R2* | GSM136905 |  |  |  |  |
|  | *ELiv14R3* | GSM136907 |  |  |  |  |
| **Developed Liver** | *DLiv1R1* | GSM213373 | 430A | WT liver from 3 month old littermates (all males) | GSE8599 |  |
|  | *DLiv1R2* | GSM213374 |  |  |  |  |
|  | *DLiv1R3* | GSM213375 |  |  |  |  |
|  | *DLiv2R1* | GSM171937 | 430A | WT liver from 4 to 5 month old mice, fasted for 18 h | GSE7137 |  |
|  | *DLiv2R2* | GSM171938 |  |  |  |  |
|  | *DLiv2R3* | GSM171939 |  |  |  |  |
|  | *DLiv2R4* | GSM171940 |  |  |  |  |
|  | *DLiv3R1* | GSM121554 | 430A | Liver from 6 week old WT mice | GSE5348 |  |
|  | *DLiv3R2* | GSM121555 |  |  |  |  |
|  | *DLiv3R3* | GSM121556 |  |  |  |  |
|  | *DLiv4R1* | GSM40065 | 430A | Livers from mice 3 months old | GSE2198 |  |
|  | *DLiv4R2* | GSM40066 |  |  |  |  |
|  | *DLiv4R3* | GSM40067 |  |  |  |  |
|  | *DLiv4R4* | GSM40068 |  |  |  |  |
|  | *DLiv5R1* | GSM44308 | 430A | Livers from 2 month old mice with Crp-lox but not activated (control mice with normal Crp levels) | GSE2362 |  |
|  | *DLiv5R2* | GSM44707 |  |  |  |  |
|  | *DLiv5R3* | GSM44708 |  |  |  |  |
|  | *DLiv6R1* | GSM185015 | 430A | Livers from diet-induced-obese mice and injected with a vehicle | GSE7648 |  |
|  | *DLiv6R2* | GSM185016 |  |  |  |  |
|  | *DLiv6R3* | GSM185017 |  |  |  |  |
|  | *DLiv6R4* | GSM185018 |  |  |  |  |
|  | *DLiv6R5* | GSM185019 |  |  |  |  |
|  | *DLiv7R1* | GSM240694 | 430A | Livers from WT mice on a normal diet | GSE9484 |  |
|  | *DLiv7R2* | GSM240695 |  |  |  |  |
|  | *DLiv7R3* | GSM240696 |  |  |  |  |
|  | *DLiv8R1* | GSM240697 | 430A | Livers from WT mice on a high fat diet |  |  |
|  | *DLiv8R2* | GSM240698 |  |  |  |  |
|  | *DLiv8R3* | GSM240699 |  |  |  |  |
|  | *DLiv8R4* | GSM240700 |  |  |  |  |
|  | *DLiv9R1* | GSM96885 | 430A | Livers from WT male mice 6-8 weeks old | GSE4262 |  |
|  | *DLiv9R2* | GSM96886 |  |  |  |  |
|  | *DLiv9R3* | GSM96887 |  |  |  |  |
|  | *DLiv10R1* | GSM154968 | 430A | Liver from 9-12 w/o WT mice fed 5% DMSO in cornoil vehicle | GSE6721 |  |
|  | *DLiv10R2* | GSM154969 |  |  |  |  |
|  | *DLiv10R3* | GSM154970 |  |  |  |  |
|  | *DLiv10R4* | GSM154971 |  |  |  |  |
|  | *DLiv11R1* | GSM138289 | 430 2.0 | Livers from WT female mice 8 weeks of age | GSE5959 |  |
|  | *DLiv11R2* | GSM138290 |  |  |  |  |
|  | *DLiv11R3* | GSM138291 |  |  |  |  |
|  | *DLiv12R1* | GSM205766 | 430 2.0 | Livers from WT male mice injected with a vehicle substance 3-5 months of age | GSE8292 |  |
|  | *DLiv12R2* | GSM205767 |  |  |  |  |
|  | *DLiv12R3* | GSM205768 |  |  |  |  |
|  | *DLiv13R1* | GSM228786 | 430 2.0 | Livers from WT mice 20 months old | GSE9012 |  |
|  | *DLiv13R2* | GSM228787 |  |  |  |  |
|  | *DLiv13R3* | GSM228788 |  |  |  |  |
|  | *DLiv13R4* | GSM228789 |  |  |  |  |
|  | *DLiv13R5* | GSM228790 |  |  |  |  |
|  | *DLiv14R1* | GSM143367 | 430 2.0 | Livers from WT mice | GSE6210 |  |
|  | *DLiv14R2* | GSM143368 |  |  |  |  |
|  | *DLiv14R3* | GSM143384 |  |  |  |  |
|  | *DLiv15R1* | GSM115690 | 430 2.0 | Livers from mice 18 weeks old fed rodent chow | GSE5128 |  |
|  | *DLiv15R2* | GSM115691 |  |  |  |  |
|  | *DLiv15R3* | GSM115692 |  |  |  |  |
|  | *DLiv16R1* | GSM115687 | 430 2.0 | Livers from mice 18 weeks old fed corn oil by gavage treatment |  |  |
|  | *DLiv16R2* | GSM115688 |  |  |  |  |
|  | *DLiv16R3* | GSM115689 |  |  |  |  |
|  | *DLiv17R1* | GSM88882 | 430 2.0 | Livers from WT mice fed a very low fat diet | GSE3889 |  |
|  | *DLiv17R2* | GSM88883 |  |  |  |  |
|  | *DLiv17R3* | GSM88884 |  |  |  |  |
|  | *DLiv17R4* | GSM88885 |  |  |  |  |
|  | *DLiv17R5* | GSM88886 |  |  |  |  |
|  | *DLiv18R1* | GSM88887 | 430 2.0 | Livers from WT mice fed a chow diet |  |  |
|  | *DLiv18R2* | GSM88888 |  |  |  |  |
|  | *DLiv18R3* | GSM88889 |  |  |  |  |
|  | *DLiv18R4* | GSM88890 |  |  |  |  |
|  | *DLiv18R5* | GSM88891 |  |  |  |  |
|  | *DLiv19R1* | GSM68933 | 430 2.0 | Livers from 4 month old WT mice | GSM68923 |  |
|  | *DLiv19R2* | GSM68934 |  |  |  |  |
|  | *DLiv19R3* | GSM68935 |  |  |  |  |
|  | *DLiv19R4* | GSM68936 |  |  |  |  |
|  | *DLiv19R5* | GSM68937 |  |  |  |  |
|  | *DLiv20R1* | GSM68923 | 430 2.0 | Livers from 10 month old WT mice |  |  |
|  | *DLiv20R2* | GSM68924 |  |  |  |  |
|  | *DLiv20R3* | GSM68925 |  |  |  |  |
|  | *DLiv20R4* | GSM68926 |  |  |  |  |
|  | *DLiv20R5* | GSM68927 |  |  |  |  |
|  | *DLiv21R1* | GSM68912 | 430 2.0 | Livers from 22 month old WT mice |  |  |
|  | *DLiv21R2* | GSM68913 |  |  |  |  |
|  | *DLiv21R3* | GSM68914 |  |  |  |  |
|  | *DLiv21R4* | GSM68915 |  |  |  |  |
|  | *DLiv21R5* | GSM68916 |  |  |  |  |
|  | *DLiv21R6* | GSM68917 |  |  |  |  |
|  | *DLiv22R1* | GSM300676 | 430 2.0 | Livers from 1 month old WT mice, RNA extracted using a miRNA kit | GSE11899 |  |
|  | *DLiv22R2* | GSM300677 |  |  |  |  |
|  | *DLiv22R3* | GSM300678 |  |  |  |  |
|  | *DLiv22R4* | GSM300679 |  |  |  |  |
|  | *DLiv22R5* | GSM300680 |  |  |  |  |
|  | *DLiv23R1* | GSM159279 | 430 2.0 | Livers from 4 to 8 week old mice fed on a high fat diet for 2 weeks | GSE6903 |  |
|  | *DLiv23R2* | GSM159280 |  |  |  |  |
|  | *DLiv23R3* | GSM159281 |  |  |  |  |
|  | *DLiv24R1* | GSM254871 | 430 2.0 | Liver samples from 15 week old mice fed corn oil through gavage | GSE10082 |  |
|  | *DLiv24R2* | GSM254877 |  |  |  |  |
|  | *DLiv24R3* | GSM254878 |  |  |  |  |
|  | *DLiv24R4* | GSM254881 |  |  |  |  |
|  | *DLiv24R5* | GSM254885 |  |  |  |  |
|  | *DLiv25R1* | GSM318909 | 430 2.0 | WT liver from adult mice | GSE12712 |  |
|  | *DLiv25R2* | GSM318910 |  |  |  |  |
|  | *DLiv25R3* | GSM318911 |  |  |  |  |
|  | *DLiv25R4* | GSM318912 |  |  |  |  |
|  | *DLiv26R1* | GSM276436 | 430 2.0 | Livers from 4-7 month old mice Genotype: B6.Pdss2loxP/loxP | GSE10904 |  |
|  | *DLiv26R2* | GSM276437 |  |  |  |  |
|  | *DLiv26R3* | GSM276438 |  |  |  |  |
|  | *DLiv27R1* | GSM177149 | 430 2.0 | Livers from WT mice 7 weeks old Strain: C57BL/6 | GSE7357 |  |
|  | *DLiv27R2* | GSM177150 |  |  |  |  |
|  | *DLiv27R3* | GSM177151 |  |  |  |  |
|  | *DLiv28R1* | GSM177155 | 430 2.0 | Livers from WT mice 7 weeks old Strain: C57BL/6 |  |  |
|  | *DLiv28R2* | GSM177156 |  |  |  |  |
|  | *DLiv28R3* | GSM177157 |  |  |  |  |
|  | *DLiv29R1* | GSM269536 | 430 2.0 | Livers of 1 month old WT mice undergone sham operation | GSE10657 |  |
|  | *DLiv29R2* | GSM269537 |  |  |  |  |
|  | *DLiv29R3* | GSM269538 |  |  |  |  |
|  | *DLiv30R1* | GSM227410 | 430 2.0 | Livers of 10 week old WT mice strain: C57B/SV129 | GSE8969 |  |
|  | *DLiv30R2* | GSM227411 |  |  |  |  |
|  | *DLiv30R3* | GSM227412 |  |  |  |  |
|  | *DLiv31R1* | GSM144725 | 430 2.0 | Livers from 8 week old mice fed a pellet diet, Strain: NMR-1 | GSE6297 |  |
|  | *DLiv31R2* | GSM144726 |  |  |  |  |
|  | *DLiv31R3* | GSM144727 |  |  |  |  |
|  | *DLiv32R1* | GSM144728 | 430 2.0 | Livers from 8 week old mice fed a pellet diet, Strain: NMR-1 |  |  |
|  | *DLiv32R2* | GSM144729 |  |  |  |  |
|  | *DLiv32R3* | GSM144730 |  |  |  |  |
|  | *DLiv33R1* | GSM252074 | 430 2.0 | Liver from 10-12 week old WT mice | GSE9954 |  |
|  | *DLiv33R2* | GSM252075 |  |  |  |  |
|  | *DLiv33R3* | GSM252076 |  |  |  |  |
|  | *DLiv34R1* | GSM102084 | 430A | (Precancerous stage) Liver samples of Mdr-2 KO heterozygous mouse 12 months old | GSE4612 |  |
|  | *DLiv34R2* | GSM102085 |  |  |  |  |
|  | *DLiv34R3* | GSM102086 |  |  |  |  |
|  | *DLiv35R1* | GSM102093 | 430A | (Precancerous stage) Liver samples of Mdr-2 KO homozygous mouse 3 months old |  |  |
|  | *DLiv35R2* | GSM102094 |  |  |  |  |
|  | *DLiv35R3* | GSM102095 |  |  |  |  |
|  | *DLiv36R1* | GSM214136 | 430A | Liver sample of Mdr-2 KO heterozygote from a mouse 16 months old | GSE8642 |  |
|  | *DLiv36R2* | GSM214137 |  |  |  |  |
|  | *DLiv36R3* | GSM214138 |  |  |  |  |
|  | *DLiv37R1* | GSM214124 | 430A | Non-tumourous liver sample from Mdr-2 KO homozygous mice 16 months old |  |  |
|  | *DLiv37R2* | GSM214125 |  |  |  |  |
|  | *DLiv37R3* | GSM214126 |  |  |  |  |
|  | *DLiv37R4* | GSM214127 |  |  |  |  |
|  | *DLiv37R5* | GSM214128 |  |  |  |  |
|  | *DLiv37R6* | GSM214129 |  |  |  |  |
| **Hepatocellular** | *HCC1R1* | GSM214130 | 430A | Liver tumour sample from Mdr-2 KO homozygous mice 16 months old | GSE8642 |  |
| **Carcinoma** | *HCC1R2* | GSM214131 |  |  |  |  |
|  | *HCC1R3* | GSM214132 |  |  |  |  |
|  | *HCC1R4* | GSM214133 |  |  |  |  |
|  | *HCC1R5* | GSM214134 |  |  |  |  |
|  | *HCC1R6* | GSM214135 |  |  |  |  |
|  | *HCC2R1* | GSM37388 | 430A | Txnip deficient mice with hepatocellular carcinoma (22 months) | GSE2127 |  |
|  | *HCC2R2* | GSM37389 |  |  |  |  |
|  | *HCC2R3* | GSM37390 |  |  |  |  |
|  | *HCC3R1* | GSM37394 | 430A | Txnip deficient mice with hepatocellular carcinoma (24 months) |  |  |
|  | *HCC3R2* | GSM37395 |  |  |  |  |
|  | *HCC3R3* | GSM37396 |  |  |  |  |
|  | *HCC4R1* | GSM37400 | 430A | Txnip deficient mice with hepatocellular carcinoma (27 months) |  |  |
|  | *HCC4R2* | GSM37401 |  |  |  |  |
|  | *HCC4R3* | GSM37402 |  |  |  |  |
|  | *HCC5R1* | GSM228781 | 430 2.0 | HCC tumour from a Trim24(TIF1a) null mice 20 months of age |  |  |
|  | *HCC5R2* | GSM228782 |  |  |  |  |
|  | *HCC5R3* | GSM228783 |  |  |  |  |
|  | *HCC5R4* | GSM228784 |  |  |  |  |
|  | *HCC5R5* | GSM228785 |  |  |  |  |
| **Embryonic Stem Cells** | *ESC1R1* | GSM86112 | 430A | J1 Embryonic stem cells differentiating into embroid bodies 0 h after inducing differentiation | GSE3749 |  |
|  | *ESC1R2* | GSM86114 |  |  |  |  |
|  | *ESC1R3* | GSM86116 |  |  |  |  |
|  | *ESC2R1* | GSM86118 | 430A | J1 Embryonic stem cells differentiating into embroid bodies 6 h after inducing differentiation |  |  |
|  | *ESC2R2* | GSM86120 |  |  |  |  |
|  | *ESC2R3* | GSM86122 |  |  |  |  |
|  | *ESC3R1* | GSM86124 | 430A | J1 Embryonic stem cells differentiating into embroid bodies 12 h after inducing differentiation |  |  |
|  | *ESC3R2* | GSM86126 |  |  |  |  |
|  | *ESC3R3* | GSM86128 |  |  |  |  |
|  | *ESC4R1* | GSM86294 | 430A | J1 Embryonic stem cells differentiating into embroid bodies 18 h after inducing differentiation |  |  |
|  | *ESC4R2* | GSM86296 |  |  |  |  |
|  | *ESC4R3* | GSM86298 |  |  |  |  |
|  | *ESC5R1* | GSM86300 | 430A | J1 Embryonic stem cells differentiating into embroid bodies 24 h after inducing differentiation |  |  |
|  | *ESC5R2* | GSM86302 |  |  |  |  |
|  | *ESC5R3* | GSM86304 |  |  |  |  |
|  | *ESC6R1* | GSM86130 | 430A | J1 Embryonic stem cells differentiating into embroid bodies 36 h after inducing differentiation |  |  |
|  | *ESC6R2* | GSM86132 |  |  |  |  |
|  | *ESC6R3* | GSM86134 |  |  |  |  |
|  | *ESC7R1* | GSM86160 | 430A | J1 Embryonic stem cells differentiating into embroid bodies 48 h after inducing differentiation |  |  |
|  | *ESC7R2* | GSM86162 |  |  |  |  |
|  | *ESC7R3* | GSM86164 |  |  |  |  |
|  | *ESC8R1* | GSM86136 | 430A | J1 Embryonic stem cells differentiating into embroid bodies 4 d after inducing differentiation |  |  |
|  | *ESC8R2* | GSM86138 |  |  |  |  |
|  | *ESC8R3* | GSM86140 |  |  |  |  |
|  | *ESC9R1* | GSM86142 | 430A | J1 Embryonic stem cells differentiating into embroid bodies 7 d after inducing differentiation |  |  |
|  | *ESC9R2* | GSM86144 |  |  |  |  |
|  | *ESC9R3* | GSM86146 |  |  |  |  |
|  | *ESC10R1* | GSM86148 | 430A | J1 Embryonic stem cells differentiating into embroid bodies 9 d after inducing differentiation |  |  |
|  | *ESC10R2* | GSM86150 |  |  |  |  |
|  | *ESC10R3* | GSM86152 |  |  |  |  |
|  | *ESC11R1* | GSM86154 | 430A | J1 Embryonic stem cells differentiating into embroid bodies 14 d after inducing differentiation |  |  |
|  | *ESC11R2* | GSM86156 |  |  |  |  |
|  | *ESC11R3* | GSM86158 |  |  |  |  |
|  | *ESC12R1* | GSM86589 | 430A | ESC grown three days in an embryonic stem cell maintenance serum | GSE3774 |  |
|  | *ESC12R2* | GSM86591 |  |  |  |  |
|  | *ESC12R3* | GSM86593 |  |  |  |  |
|  | *ESC13R1* | GSM86595 | 430A | ESC grown three days in a serum that failed all tests for embryonic stem cell maintenance |  |  |
|  | *ESC13R2* | GSM86597 |  |  |  |  |
|  | *ESC13R3* | GSM86599 |  |  |  |  |
|  | *ESC14R1* | GSM72616 | 430A | Embryonic bodies | GSE3223 |  |
|  | *ESC14R2* | GSM72618 |  |  |  |  |
|  | *ESC14R3* | GSM72621 |  |  |  |  |
|  | *ESC15R1* | GSM72622 | 430A | Embryonic stem cells |  |  |
|  | *ESC15R2* | GSM72624 |  |  |  |  |
|  | *ESC15R3* | GSM72626 |  |  |  |  |
|  | *ESC16R1* | GSM64922 | 430A | R1 Embryonic stem cells differentiating into embroid bodies 0 h after inducing differentiation | GSE2972 |  |
|  | *ESC16R2* | GSM64924 |  |  |  |  |
|  | *ESC16R3* | GSM64926 |  |  |  |  |
|  | *ESC17R1* | GSM64928 | 430A | R1 Embryonic stem cells differentiating into embroid bodies 6 h after inducing differentiation |  |  |
|  | *ESC17R2* | GSM64930 |  |  |  |  |
|  | *ESC17R3* | GSM64932 |  |  |  |  |
|  | *ESC18R1* | GSM64934 | 430A | R1 Embryonic stem cells differentiating into embroid bodies 12 h after inducing differentiation |  |  |
|  | *ESC18R2* | GSM64936 |  |  |  |  |
|  | *ESC18R3* | GSM64938 |  |  |  |  |
|  | *ESC19R1* | GSM64940 | 430A | R1 Embryonic stem cells differentiating into embroid bodies 18 h after inducing differentiation |  |  |
|  | *ESC19R2* | GSM64942 |  |  |  |  |
|  | *ESC19R3* | GSM64944 |  |  |  |  |
|  | *ESC20R1* | GSM64946 | 430A | R1 Embryonic stem cells differentiating into embroid bodies 24 h after inducing differentiation |  |  |
|  | *ESC20R2* | GSM64948 |  |  |  |  |
|  | *ESC20R3* | GSM64950 |  |  |  |  |
|  | *ESC21R1* | GSM64952 | 430A | R1 Embryonic stem cells differentiating into embroid bodies 36 h after inducing differentiation |  |  |
|  | *ESC21R2* | GSM64954 |  |  |  |  |
|  | *ESC21R3* | GSM64956 |  |  |  |  |
|  | *ESC22R1* | GSM64958 | 430A | R1 Embryonic stem cells differentiating into embroid bodies 48 h after inducing differentiation |  |  |
|  | *ESC22R2* | GSM64960 |  |  |  |  |
|  | *ESC22R3* | GSM64962 |  |  |  |  |
|  | *ESC23R1* | GSM64964 | 430A | R1 Embryonic stem cells differentiating into embroid bodies 4 d after inducing differentiation |  |  |
|  | *ESC23R2* | GSM64966 |  |  |  |  |
|  | *ESC23R3* | GSM64968 |  |  |  |  |
|  | *ESC24R1* | GSM64970 | 430A | R1 Embryonic stem cells differentiating into embroid bodies 7 d after inducing differentiation |  |  |
|  | *ESC24R2* | GSM64972 |  |  |  |  |
|  | *ESC24R3* | GSM64974 |  |  |  |  |
|  | *ESC25R1* | GSM64976 | 430A | R1 Embryonic stem cells differentiating into embroid bodies 9 d after inducing differentiation |  |  |
|  | *ESC25R2* | GSM64978 |  |  |  |  |
|  | *ESC25R3* | GSM64980 |  |  |  |  |
|  | *ESC26R1* | GSM64982 | 430A | R1 Embryonic stem cells differentiating into embroid bodies 14 d after inducing differentiation |  |  |
|  | *ESC26R2* | GSM64984 |  |  |  |  |
|  | *ESC26R3* | GSM64986 |  |  |  |  |
|  | *ESC27R1* | GSM201479 | 430 2.0 | Mouse E14 Embryonic stem cells | GSE8128 |  |
|  | *ESC27R2* | GSM201482 |  |  |  |  |
|  | *ESC27R3* | GSM201484 |  |  |  |  |
|  | *ESC27R4* | GSM201486 |  |  |  |  |
|  | *ESC28R1* | GSM201478 | 430 2.0 | Embroid bodies |  |  |
|  | *ESC28R2* | GSM201480 |  |  |  |  |
|  | *ESC28R3* | GSM201481 |  |  |  |  |
|  | *ESC28R4* | GSM201483 |  |  |  |  |
|  | *ESC28R5* | GSM201485 |  |  |  |  |
|  | *ESC29R1* | GSM271925 | 430 2.0 | Embryonic stem cells derived from parthenogenesis | GSE10776 |  |
|  | *ESC29R2* | GSM271926 |  |  |  |  |
|  | *ESC29R3* | GSM271927 |  |  |  |  |
|  | *ESC30R1* | GSM272032 | 430 2.0 | Embryonic stem cells derived from parthenogenesis |  |  |
|  | *ESC30R2* | GSM272033 |  |  |  |  |
|  | *ESC30R3* | GSM272034 |  |  |  |  |
|  | *ESC31R1* | GSM272035 | 430 2.0 | Embryonic stem cells derived from normal fertilisation |  |  |
|  | *ESC31R2* | GSM272036 |  |  |  |  |
|  | *ESC31R3* | GSM272037 |  |  |  |  |
|  | *ESC32R1* | GSM272049 | 430 2.0 | Embryonic stem cells derived from parthenogenesis |  |  |
|  | *ESC32R2* | GSM272050 |  |  |  |  |
|  | *ESC32R3* | GSM272051 |  |  |  |  |
|  | *ESC33R1* | GSM272052 | 430 2.0 | Embryonic stem cells derived from ATCC |  |  |
|  | *ESC33R2* | GSM272053 |  |  |  |  |
|  | *ESC33R3* | GSM272054 |  |  |  |  |
|  | *ESC34R1* | GSM304953 | 430 2.0 | ESCs with strain Oct4-Gip passage 25 |  |  |
|  | *ESC34R2* | GSM304954 |  |  |  |  |
|  | *ESC34R3* | GSM304955 |  |  |  |  |
|  | *ESC34R4* | GSM304957 |  |  |  |  |
|  | *ESC35R1* | GSM241871 | 430 2.0 | ESCs R-1, Undifferentiated Mouse Embryonic stem cells | GSE9563 |  |
|  | *ESC35R2* | GSM241872 |  |  |  |  |
|  | *ESC35R3* | GSM241873 |  |  |  |  |
|  | *ESC36R1* | GSM241874 | 430 2.0 | R-1, Differentiated day-5 Embryoid bodies from mouse ESCs |  |  |
|  | *ESC36R2* | GSM241875 |  |  |  |  |
|  | *ESC36R3* | GSM241876 |  |  |  |  |
| **Muscle cell lines** | *Musc1R1* | GSM72628 | 430A | C2C12 myoblasts transfected with an empty vector | GSE3224 |  |
|  | *Musc1R2* | GSM72630 |  |  |  |  |
|  | *Musc1R3* | GSM72632 |  |  |  |  |
|  | *Musc2R1* | GSM16840 | 430A | Extraocular muscle cell line from WT mice | GSE4463 |  |
|  | *Musc2R2* | GSM16841 |  |  |  |  |
|  | *Musc2R3* | GSM16842 |  |  |  |  |
|  | *Musc3R1* | GSM16855 | 430A | Hind leg cell line from WT mice |  |  |
|  | *Musc3R2* | GSM16856 |  |  |  |  |
|  | *Musc3R3* | GSM16857 |  |  |  |  |
|  | *Musc4R1* | GSM119559 | 430A | C2C12 cells in growth medium transfected with an empty vector | GSE5305 |  |
|  | *Musc4R2* | GSM119563 |  |  |  |  |
|  | *Musc4R3* | GSM119565 |  |  |  |  |
|  | *Musc5R1* | GSM119558 | 430A | C2C12 cells in differentiation medium transfected with an empty vector |  |  |
|  | *Musc5R2* | GSM119568 |  |  |  |  |
|  | *Musc5R3* | GSM119571 |  |  |  |  |
|  | *Musc6R1* | GSM30759 | 430A | C2C12 myoblasts in differentiation medium | GSE1776 |  |
|  | *Musc6R2* | GSM30771 |  |  |  |  |
|  | *Musc6R3* | GSM30772 |  |  |  |  |
|  | *Musc6R4* | GSM30773 |  |  |  |  |
|  | *Musc7R1* | GSM30774 | 430A | Starved C2C12 myoblasts in differentiation medium |  |  |
|  | *Musc7R2* | GSM30775 |  |  |  |  |
|  | *Musc7R3* | GSM30776 |  |  |  |  |
|  | *Musc7R4* | GSM30777 |  |  |  |  |
|  | *Musc8R1* | GSM288011 | 430 2.0 | C2C12 myoblasts at 50% confluency | GSE11415 |  |
|  | *Musc8R2* | GSM288012 |  |  |  |  |
|  | *Musc8R3* | GSM288013 |  |  |  |  |
|  | *Musc9R1* | GSM288014 | 430 2.0 | C2C12 myoblasts at 90% confluency |  |  |
|  | *Musc9R2* | GSM288015 |  |  |  |  |
|  | *Musc9R3* | GSM288016 |  |  |  |  |
|  | *Musc10R1* | GSM288017 | 430 2.0 | C2C12 myotube differentiation 6 h |  |  |
|  | *Musc10R2* | GSM288018 |  |  |  |  |
|  | *Musc10R3* | GSM288019 |  |  |  |  |
|  | *Musc11R1* | GSM288020 | 430 2.0 | C2C12 myotube differentiation Day 1 |  |  |
|  | *Musc11R2* | GSM288021 |  |  |  |  |
|  | *Musc11R3* | GSM288022 |  |  |  |  |
|  | *Musc12R1* | GSM288023 | 430 2.0 | C2C12 myotube differentiation Day 2 |  |  |
|  | *Musc12R2* | GSM288024 |  |  |  |  |
|  | *Musc12R3* | GSM288025 |  |  |  |  |
|  | *Musc13R1* | GSM288026 | 430 2.0 | C2C12 myotube differentiation Day 3 |  |  |
|  | *Musc13R2* | GSM288027 |  |  |  |  |
|  | *Musc13R3* | GSM288028 |  |  |  |  |
|  | *Musc14R1* | GSM288029 | 430 2.0 | C2C12 myotube differentiation Day 4 |  |  |
|  | *Musc14R2* | GSM288030 |  |  |  |  |
|  | *Musc14R3* | GSM288031 |  |  |  |  |
|  | *Musc15R1* | GSM288032 | 430 2.0 | C2C12 myotube differentiation Day 5 |  |  |
|  | *Musc15R2* | GSM288033 |  |  |  |  |
|  | *Musc15R3* | GSM288034 |  |  |  |  |

**Supplementary** **Table 2** – Primer Sequences

| **Gene** | **Forward Primer Sequence** | **Reverse Primer Sequence** | **Universal Probe Library Probe #** |
| --- | --- | --- | --- |
| Taf4a | ccacagcagatccaactgaa | ggtaacacggtgggtttcac | 71 |
| Murc | gtcgaaaccaagcaagaagaa | ggcaggcttctgtctttaaca | 79 |
| Mcm2 | ctcagaatcaggaggtgaagc | gcggatacgttggtagttctg | 3 |
| Ltbp3 | ttccgggttcttaccgttg | tcccagcctcacactcatc | 104 |
| Anxa13 | tgtacaaagcctgcaaagga | tgctttatctgttgcctctcc | 18 |
| Sox9 | gaagctggcagaccagtacc | ggtctcttctcgctctcgttc | 75 |
| CD24a | cttctggcactgctcctacc | tggtggtagcgttacttgga | 38 |
| Ncam | gtggtatgatgccaaagaagc | ccgagtacctcgtctcaggt | 97 |
| M2pk | aagggggactaccctctgg | cctcgaatagctgcaagtgg | 34 |

**Supplementary Table 3.** Cluster A probe sets, their corresponding gene names/symbols and expression fold changes between liver progenitor cells (LPC)/developed liver (DL). Probe sets are grouped by pathway in order of statistical significance, as determined by Database for Annotation, Visualization and Integrated Discovery and KEGG pathway analysis.

| **MAPK** | **Gene name** | **Gene symbol** | **Fold-change (LPC/DL)** |
| --- | --- | --- | --- |
| 1417268_at | CD14 antigen | Cd14 | 22.00 |
| 1426478_at | RAS p21 protein activator 1 | Rasa1 | 3.11 |
| 1422785_at | RAS p21 protein activator 2 | Rasa2 | 8.41 |
| 1451233_at | TNF receptor-associated factor 2 | Traf2 | 9.58 |
| 1435350_at | TNF receptor-associated factor 6 | Traf6 | 2.42 |
| 1422168_a_at, 1422169_a_at | brain derived neurotrophic factor | Bdnf | 59.59, Not expressed in DL |
| 1448656_at | calcium channel, voltage-dependent, beta 3 subunit | Cacnb3 | Not expressed in DL |
| 1417164_at | dual specificity phosphatase 10 | Dusp10 | 16.80 |
| 1418497_at | fibroblast growth factor 13 | Fgf13 | Not expressed in DL |
| 1426233_at | mitogen-activated protein kinase kinase 4 | Map2k4 | 1.35 |
| 1426850_a_at | mitogen-activated protein kinase kinase 6 | Map2k6 | 29.26 |
| 1422615_at, 1448050_s_at | mitogen-activated protein kinase kinase kinase kinase 4 | Map4k4 | 29.61, Not expressed in DL |
| 1450070_s_at | p21 protein (Cdc42/Rac)-activated kinase 1 | Pak1 | 26.99 |
| 1425045_at | phospholipase A2, group IVB (cytosolic); jumonji domain containing 7 | Jmjd7 /// Pla2g4b | 16.94 |
| 1450945_at | protein kinase C, alpha | Prkca | 93.13 |
| 1451299_at | protein kinase, X-linked | Prkx | 4.20 |
| 1430025_at | protein phosphatase 3, catalytic subunit, gamma isoform | Ppp3cc | 14.11 |
| 1427299_at | ribosomal protein S6 kinase polypeptide 3 | Rps6ka3 | 195.65 |
| 1448498_at | ribosomal protein S6 kinase, polypeptide 4 | Rps6ka4 | 78.07 |
| 1418513_at | serine/threonine kinase 3 (Ste20, yeast homolog) | Stk3 | 7.39 |
| 1450414_at | similar to platelet-derived growth factor B chain; platelet derived growth factor, B polypeptide | Pdgfb | Not expressed in DL |
| 1421884_at | son of sevenless homolog 1 (Drosophila) | Sos1 | 28.13 |
| 1448113_at | stathmin 1; predicted gene 11223; predicted gene 6393 | Gm11223 /// Stmn1 | 56.08 |
| 1420653_at | transforming growth factor, beta 1 | Tgfb1 | 13.24 |
| 1450922_a_at | transforming growth factor, beta 2 | Tgfb2 | Not expressed in DL |
|  |  |  |  |
| **GnRH** | **Gene Name** | **Gene Symbol** | **Fold-change (LPC/DL)** |
| 1434653_at | PTK2 protein tyrosine kinase 2 beta | Ptk2b | 1.59 |
| 1455462_at | adenylate cyclase 2 | Adcy2 | 1.08 |
| 1422659_at, 1427763_a_at | calcium/calmodulin-dependent protein kinase II, delta | Camk2d | 17.47, 10.43 |
| 1418349_at | heparin-binding EGF-like growth factor | Hbegf | 76.82 |
| 1426233_at | mitogen-activated protein kinase kinase 4 | Map2k4 | 1.35 |
| 1426850_a_at | mitogen-activated protein kinase kinase 6 | Map2k6 | 29.26 |
| 1425045_at | phospholipase A2, group IVB (cytosolic); jumonji domain containing 7 | Jmjd7 /// Pla2g4b | 16.94 |
| 1437113_s_at | phospholipase D1 | Pld1 | 2.61 |
| 1450945_at | protein kinase C, alpha | Prkca | 93.13 |
| 1451299_at | protein kinase, X-linked | Prkx | 4.20 |
| 1423941_at | similar to Calcium/calmodulin-dependent protein kinase type II gamma chain (CaM-kinase II gamma chain) (CaM kinase II gamma subunit) (CaMK-II subunit gamma); calcium/calmodulin-dependent protein kinase II gamma | Camk2g | 7.18 |
| 1421884_at | son of sevenless homolog 1 (Drosophila) | Sos1 | 28.13 |
|  |  |  |  |
| **Cell Cycle** | **Gene Name** | **Gene Symbol** | **Fold-change (LPC/DL)** |
| 1421205_at | ataxia telangiectasia mutated homolog (human) | Atm | 1.98 |
| 1438571_at | budding uninhibited by benzimidazoles 1 homolog (S. cerevisiae) | Bub1 | 43.66 |
| 1423999_at | c-abl oncogene 1, receptor tyrosine kinase | Abl1 | 8.33 |
| 1422252_a_at | cell division cycle 25 homolog C (S. pombe) | Cdc25c | Not expressed in DL |
| 1449708_s_at | checkpoint kinase 1 homolog (S. pombe) | Chek1 | 113.35 |
| 1416124_at | cyclin D2 | Ccnd2 | Not expressed in DL |
| 1422535_at | cyclin E2 | Ccne2 | 5.95 |
| 1450951_at | predicted gene 8892; structural maintenace of chromosomes 3 | Smc3 | Not expressed in DL |
| 1451576_at | protein kinase, DNA activated, catalytic polypeptide | Prkdc | 6.87 |
| 1425166_at | retinoblastoma-like 1 (p107) | Rbl1 | 44.21 |
| 1438623_x_at | ring-box 1; predicted gene 9840 | Gm9840 /// Rbx1 | 6.59 |
| 1449152_at | similar to Cyclin-dependent kinase 4 inhibitor B (p14-INK4b) (p15-INK4b); cyclin-dependent kinase inhibitor 2B (p15, inhibits CDK4) | Cdkn2b | Not expressed in DL |
| 1420653_at | transforming growth factor, beta 1 | Tgfb1 | 13.24 |
| 1450922_a_at | transforming growth factor, beta 2 | Tgfb2 | Not expressed in DL |
|  |  |  |  |
| **ErbB** | **Gene Name** | **Gene Symbol** | **Fold-change (LPC/DL)** |
| 1421134_at | amphiregulin | Areg | Not expressed in DL |
| 1421161_at, 1435541_at | betacellulin, epidermal growth factor family member | Btc | 33.83, 4.71 |
| 1423999_at | c-abl oncogene 1, receptor tyrosine kinase | Abl1 | 8.33 |
| 1422659_at, 1427763_a_at | calcium/calmodulin-dependent protein kinase II, delta | Camk2d | 17.47, 10.43 |
| 1418349_at | heparin-binding EGF-like growth factor | Hbegf | 76.82 |
| 1426233_at | mitogen-activated protein kinase kinase 4 | Map2k4 | 1.35 |
| 1450070_s_at | p21 protein (Cdc42/Rac)-activated kinase 1 | Pak1 | 26.99 |
| 1450945_at | protein kinase C, alpha | Prkca | 93.13 |
| 1428849_at | ribosomal protein S6 kinase, polypeptide 1 | Rps6kb1 | 8.86 |
| 1423941_at | similar to Calcium/calmodulin-dependent protein kinase type II gamma chain (CaM-kinase II gamma chain) (CaM kinase II gamma subunit) (CaMK-II subunit gamma); calcium/calmodulin-dependent protein kinase II gamma | Camk2g | 7.18 |
| 1421884_at | son of sevenless homolog 1 (Drosophila) | Sos1 | 28.13 |
|  |  |  |  |
| **TGF-beta** | **Gene Name** | **Gene Symbol** | **Fold-change (LPC/DL)** |
| 1423592_at | Rho-associated coiled-coil containing protein kinase 2 | Rock2 | 10.38 |
| 1451004_at | activin receptor IIA | Acvr2a | 3.31 |
| 1448870_at | latent transforming growth factor beta binding protein 1 | Ltbp1 | 127.84 |
| 1425166_at | retinoblastoma-like 1 (p107) | Rbl1 | 44.21 |
| 1428849_at | ribosomal protein S6 kinase, polypeptide 1 | Rps6kb1 | 8.86 |
| 1438623_x_at | ring-box 1; predicted gene 9840 | Gm9840 /// Rbx1 | 6.59 |
| 1449152_at | similar to Cyclin-dependent kinase 4 inhibitor B (p14-INK4b) (p15-INK4b); cyclin-dependent kinase inhibitor 2B (p15, inhibits CDK4) | Cdkn2b | Not expressed in DL |
| 1421811_at | thrombospondin 1; similar to thrombospondin 1 | Pald1 /// Thbs1 | 6.89 |
| 1420653_at | transforming growth factor, beta 1 | Tgfb1 | 13.24 |
| 1450922_a_at | transforming growth factor, beta 2 | Tgfb2 | Not expressed in DL |
| 1447947_at | zinc finger, FYVE domain containing 16 | Zfyve16 | 7.34 |
|  |  |  |  |
| **Wnt** | **Gene Name** | **Gene Symbol** | **Fold-change (LPC/DL)** |
| 1423592_at | Rho-associated coiled-coil containing protein kinase 2 | Rock2 | 10.38 |
| 1422659_at, 1427763_a_at | calcium/calmodulin-dependent protein kinase II, delta | Camk2d | 17.47, 10.43 |
| 1460646_at | casein kinase 2, alpha prime polypeptide; similar to casein kinase II, alpha prime subunit | Csnk2a2 | 14.73 |
| 1416124_at | cyclin D2 | Ccnd2 | Not expressed in DL |
| 1417487_at | fos-like antigen 1 | Fosl1 | Not expressed in DL |
| 1418534_at | frizzled homolog 2 (Drosophila) | Fzd2 | Not expressed in DL |
| 1417301_at | frizzled homolog 6 (Drosophila) | Fzd6 | 48.95 |
| 1449299_at | low density lipoprotein receptor-related protein 5 | Lrp5 | 2.67 |
| 1450945_at | protein kinase C, alpha | Prkca | 93.13 |
| 1451299_at | protein kinase, X-linked | Prkx | 4.20 |
| 1430025_at | protein phosphatase 3, catalytic subunit, gamma isoform | Ppp3cc | 14.11 |
| 1438623_x_at | ring-box 1; predicted gene 9840 | Gm9840 /// Rbx1 | 6.59 |
| 1420106_at, 1423390_at | seven in absentia 1A | Siah1a | 7.47, 8.09 |
| 1423941_at | similar to Calcium/calmodulin-dependent protein kinase type II gamma chain (CaM-kinase II gamma chain) (CaM kinase II gamma subunit) (CaMK-II subunit gamma); calcium/calmodulin-dependent protein kinase II gamma | Camk2g | 7.18 |
| 1455109_at | transducin (beta)-like 1X-linked receptor 1 | Tbl1xr1 | 3.19 |
|  |  |  |  |
| **Heparin sulfate biosynthesis** | **Gene Name** | **Gene Symbol** | **Fold-change (LPC/DL)** |
| 1422539_at | exostoses (multiple)-like 2 | Extl2 | 3.49 |
| 1423450_a_at | heparan sulfate (glucosamine) 3-O-sulfotransferase 1 | Hs3st1 | Not expressed in DL |
| 1450730_at, 1422739_at | heparan sulfate 2-O-sulfotransferase 1 | Hs2st1 | Not expressed in DL, 6.89 |
| 1450047_at | heparan sulfate 6-O-sulfotransferase 2 | Hs6st2 | Not expressed in DL |
| 1435252_at | similar to UDP-Gal:betaGal beta 1,3-galactosyltransferase, polypeptide 6; UDP-Gal:betaGal beta 1,3-galactosyltransferase, polypeptide 6 | B3galt6 | 18.88 |
| 1426303_at | xylosylprotein beta1,4-galactosyltransferase, polypeptide 7 (galactosyltransferase I) | B4galt7 | 8.96 |
|  |  |  |  |
| **Axon guidance** | **Gene Name** | **Gene Symbol** | **Fold-change (LPC/DL)** |
| 1421928_at | Eph receptor A4 | Epha4 | Not expressed in DL |
| 1451991_at | Eph receptor A7 | Epha7 | 56.50 |
| 1426478_at | RAS p21 protein activator 1 | Rasa1 | 3.11 |
| 1423592_at | Rho-associated coiled-coil containing protein kinase 2 | Rock2 | 10.38 |
| 1423999_at | c-abl oncogene 1, receptor tyrosine kinase | Abl1 | 8.33 |
| 1419638_at, 1419639_at | ephrin B2 | Efnb2 | 174.25, 319.51 |
| 1434440_at | guanine nucleotide binding protein (G protein), alpha inhibiting 1 | Gnai1 | 17.14 |
| 1450070_s_at | p21 protein (Cdc42/Rac)-activated kinase 1 | Pak1 | 26.99 |
| 1430025_at | protein phosphatase 3, catalytic subunit, gamma isoform | Ppp3cc | 14.11 |
| 1419061_at | ras homolog gene family, member D | Rhod | 3.45 |
| 1420696_at | sema domain, immunoglobulin domain (Ig), short basic domain, secreted, (semaphorin) 3C | Sema3c | Not expressed in DL |
| 1427673_a_at, 1425906_a_at | sema domain, immunoglobulin domain (Ig), short basic domain, secreted, (semaphorin) 3E; hypothetical protein LOC100044162 | Sema3e | Not expressed in DL, Not expressed in DL |
| 1435110_at | unc-5 homolog B (C. elegans) | Unc5b | Not expressed in DL |
|  |  |  |  |
| **Pathways in cancer** | **Gene Name** | **Gene Symbol** | **Fold-change (LPC/DL)** |
| 1416888_at | Fas (TNFRSF6)-associated via death domain | Fadd | 29.65 |
| 1451233_at | TNF receptor-associated factor 2 | Traf2 | 9.58 |
| 1435350_at | TNF receptor-associated factor 6 | Traf6 | 2.42 |
| 1423999_at | c-abl oncogene 1, receptor tyrosine kinase | Abl1 | 8.33 |
| 1419765_at | cullin 2 | Cul2 | 1.73 |
| 1422535_at | cyclin E2 | Ccne2 | 5.95 |
| 1418497_at | fibroblast growth factor 13 | Fgf13 | Not expressed in DL |
| 1418534_at | frizzled homolog 2 (Drosophila) | Fzd2 | Not expressed in DL |
| 1417301_at | frizzled homolog 6 (Drosophila) | Fzd6 | 48.95 |
| 1421198_at | integrin alpha V | Itgav | 18.47 |
| 1415854_at, 1415855_at, 1448117_at | kit ligand | Kitl | Not expressed in DL, 38.75, 154.39 |
| 1427010_s_at | laminin, alpha 5 | Lama5 | Not expressed in DL |
| 1416513_at | laminin, beta 2 | Lamb2 | 7.35 |
| 1420715_a_at | peroxisome proliferator activated receptor gamma | Pparg | 20.49 |
| 1417263_at, 1417262_at | prostaglandin-endoperoxide synthase 2 | Ptgs2 | Not expressed in DL, Not expressed in DL |
| 1455486_at | protein inhibitor of activated STAT 1 | Pias1 | 2.34 |
| 1450945_at | protein kinase C, alpha | Prkca | 93.13 |
| 1438623_x_at | ring-box 1; predicted gene 9840 | Gm9840 /// Rbx1 | 6.59 |
| 1449152_at | similar to Cyclin-dependent kinase 4 inhibitor B (p14-INK4b) (p15-INK4b); cyclin-dependent kinase inhibitor 2B (p15, inhibits CDK4) | Cdkn2b | Not expressed in DL |
| 1450414_at | similar to platelet-derived growth factor B chain; platelet derived growth factor, B polypeptide | Pdgfb | Not expressed in DL |
| 1421884_at | son of sevenless homolog 1 (Drosophila) | Sos1 | 28.13 |
| 1420653_at | transforming growth factor, beta 1 | Tgfb1 | 13.24 |
| 1450922_a_at | transforming growth factor, beta 2 | Tgfb2 | Not expressed in DL |
|  |  |  |  |
| **Oocyte meiosis** | **Gene Name** | **Gene Symbol** | **Fold-change (LPC/DL)** |
| 1455462_at | adenylate cyclase 2 | Adcy2 | 1.08 |
| 1438571_at | budding uninhibited by benzimidazoles 1 homolog (S. cerevisiae) | Bub1 | 43.66 |
| 1422659_at, 1427763_a_at | calcium/calmodulin-dependent protein kinase II, delta | Camk2d | 17.47, 10.43 |
| 1422252_a_at | cell division cycle 25 homolog C (S. pombe) | Cdc25c | Not expressed in DL |
| 1422535_at | cyclin E2 | Ccne2 | 5.95 |
| 1450951_at | predicted gene 8892; structural maintenace of chromosomes 3 | Smc3 | Not expressed in DL |
| 1451299_at | protein kinase, X-linked | Prkx | 4.20 |
| 1430025_at | protein phosphatase 3, catalytic subunit, gamma isoform | Ppp3cc | 14.11 |
| 1427299_at | ribosomal protein S6 kinase polypeptide 3 | Rps6ka3 | 195.65 |
| 1438623_x_at | ring-box 1; predicted gene 9840 | Gm9840 /// Rbx1 | 6.59 |
| 1423941_at | similar to Calcium/calmodulin-dependent protein kinase type II gamma chain (CaM-kinase II gamma chain) (CaM kinase II gamma subunit) (CaMK-II subunit gamma); calcium/calmodulin-dependent protein kinase II gamma | Camk2g | 7.18 |
|  |  |  |  |
| **p53 signaling pathway** | **Gene Name** | **Gene Symbol** | **Fold-change (LPC/DL)** |
| 1421205_at | ataxia telangiectasia mutated homolog (human) | Atm | 1.98 |
| 1449708_s_at | checkpoint kinase 1 homolog (S. pombe) | Chek1 | 113.35 |
| 1416124_at | cyclin D2 | Ccnd2 | Not expressed in DL |
| 1422535_at | cyclin E2 | Ccne2 | 5.95 |
| 1425706_a_at, 1436680_s_at | damage specific DNA binding protein 2 | Ddb2 | 1.88, 2.03 |
| 1420106_at, 1423390_at | seven in absentia 1A | Siah1a | 7.47, 8.09 |
| 1421811_at | thrombospondin 1; similar to thrombospondin 1 | Pald1 /// Thbs1 | 6.89 |
| 1419558_at | transformed mouse 3T3 cell double minute 4 | Mdm4 | 2.62 |
|  |  |  |  |
| **Small cell lung cancer** | **Gene Name** | **Gene Symbol** | **Fold-change (LPC/DL)** |
| 1451233_at | TNF receptor-associated factor 2 | Traf2 | 9.58 |
| 1435350_at | TNF receptor-associated factor 6 | Traf6 | 2.42 |
| 1422535_at | cyclin E2 | Ccne2 | 5.95 |
| 1421198_at | integrin alpha V | Itgav | 18.47 |
| 1427010_s_at | laminin, alpha 5 | Lama5 | Not expressed in DL |
| 1416513_at | laminin, beta 2 | Lamb2 | 7.35 |
| 1417263_at, 1417262_at | prostaglandin-endoperoxide synthase 2 | Ptgs2 | Not expressed in DL, Not expressed in DL |
| 1455486_at | protein inhibitor of activated STAT 1 | Pias1 | 2.34 |
| 1449152_at | similar to Cyclin-dependent kinase 4 inhibitor B (p14-INK4b) (p15-INK4b); cyclin-dependent kinase inhibitor 2B (p15, inhibits CDK4) | Cdkn2b | Not expressed in DL |
|  |  |  |  |
| **Jak-STAT signaling pathway** | **Gene Name** | **Gene Symbol** | **Fold-change (LPC/DL)** |
| 1422033_a_at | Zfp91-Cntf readthrough transcript; zinc finger protein 91; ciliary neurotrophic factor | Cntf /// U05342 /// Zfp91 | 2.06 |
| 1437271_at, 1437270_a_at | cardiotrophin-like cytokine factor 1 | Clcf1 | Not expressed in DL, Not expressed in DL |
| 1416124_at | cyclin D2 | Ccnd2 | Not expressed in DL |
| 1421034_a_at | interleukin 4 receptor, alpha | Il4ra | 1.78 |
| 1421207_at | leukemia inhibitory factor | Lif | Not expressed in DL |
| 1418674_at | oncostatin M receptor | Osmr | 5.82 |
| 1455486_at | protein inhibitor of activated STAT 1 | Pias1 | 2.34 |
| 1416862_at | signal transducing adaptor molecule (SH3 domain and ITAM motif) 1 | Stam | 4.63 |
| 1416976_at | signal transducing adaptor molecule (SH3 domain and ITAM motif) 2 | Stam2 | 27.99 |
| 1421884_at | son of sevenless homolog 1 (Drosophila) | Sos1 | 28.13 |
| 1423162_s_at | sprouty protein with EVH-1 domain 1, related sequence | Spred1 | 12.33 |
| 1421275_s_at | suppressor of cytokine signaling 4 | Socs4 | 9.03 |
| 1423350_at | suppressor of cytokine signaling 5 | Socs5 | 15.06 |
|  |  |  |  |
| **Ribosome** | **Gene Name** | **Gene Symbol** | **Fold-change (LPC/DL)** |
| 1420381_a_at | predicted gene 10191; predicted gene 7689; predicted gene 9401; similar to ribosomal protein L31; hypothetical protein LOC675768; predicted gene 13004; predicted gene 9228; predicted gene 10072; predicted gene 5437; predicted gene 9154; ribosomal protein L31; predicted gene 6670; predicted gene 8759 | LOC638399 /// Rpl31 /// Rpl31-ps12 | 39.58 |
| 1423070_at | predicted gene 12618; predicted gene 8724; predicted gene 10155; predicted gene 3355; predicted gene 3713; predicted gene 3201; predicted gene 13641; similar to ribosomal protein L21; predicted gene 12411; predicted gene 5445; predicted gene 5495; predicted gene 13604; predicted gene 10045; predicted gene 14648; predicted gene 15150; predicted gene 8252; predicted gene 8157; predicted gene 8880; predicted gene 6813; predicted gene 15312; predicted gene 5534; predicted gene 8054; predicted gene 10095; predicted gene 5857; predicted gene 8195; predicted gene 8840; predicted gene 10240; predicted gene 5810; predicted gene 11975; predicted gene 5816; predicted gene 7547; predicted gene 7702; predicted gene 8012; predicted gene 8557; predicted gene 12760; predicted gene 2815; predicted gene 14336; predicted gene 7806; predicted gene 7799; predicted gene 5042; predicted gene 7062; similar to 60S ribosomal protein L21; predicted gene 8397; predicted gene 5528; ribosomal protein L21; predicted gene 11703; predicted gene 12944; predicted gene 5502; predicted gene 10163; predicted gene 7218; predicted gene 15309; ribosomal protein L21 pseudogene; predicted gene 8915; predicted gene 16060; predicted gene 13653; predicted gene 6689; predicted gene 8101; predicted gene 9130 | Gm10045 /// Gm11703 /// Gm13653 /// Gm15682 /// Gm5445 /// Gm5528 /// LOC100505283 /// LOC634339 /// Rpl21 | 5.83 |
| 1438076_at | predicted gene 4091; predicted gene 9703; predicted gene 12191; predicted gene 7429; similar to ribosomal protein L30; predicted gene 4483; ribosomal protein L30; predicted gene 2648; predicted gene 8808; predicted gene 6570; predicted gene 6109; ribosomal protein L30, pseudogene 2 | Gm5481 /// Gm6109 /// Gm6570 /// Gm7429 /// Rpl30 | 11.25 |
| 1421935_at | predicted gene 4997; predicted gene 6440; ribosomal protein S20; predicted gene 4332; similar to 40S ribosomal protein S20; similar to ribosomal protein S20 | Rps20 | 7.68 |
| 1438527_at | predicted gene 5879; similar to 60S ribosomal protein L3 (J1 protein); predicted gene 4959; predicted gene 6035; predicted gene 12816; ribosomal protein L3; predicted gene 5851; predicted gene 16468; predicted gene 5850 | Gm12816 /// Gm5879 /// Rpl3 | 32.45 |
| 1456365_at | predicted gene 5928; predicted gene 12617; predicted gene 4802; similar to ribosomal protein S27a; predicted gene 13215; predicted gene 6111; predicted gene 7808; predicted gene 6014; predicted gene 8317; ubiquitin C; ubiquitin B; similar to fusion protein: ubiquitin (bases 43_513); ribosomal protein S27a (bases 217_532); similar to ubiquitin B; predicted gene 8649; ribosomal protein S27A; predicted gene 11517; predicted gene 11808; predicted gene 8430; RIKEN cDNA 2810422J05 gene; similar to Ubc protein; predicted gene 13815; ubiquitin A-52 residue ribosomal protein fusion product 1; predicted gene 8797; predicted gene 1821; predicted gene 11759; predicted gene 5239; predicted gene 6438 | Gm11517 | 42.65 |
| 1454833_at | ribosomal protein L35; predicted gene 10269; predicted gene 8444; predicted gene 2000; predicted gene 4342 | Rpl35 | 7.07 |
| 1426958_at | ribosomal protein S9; predicted gene 5905 | Rps9 | 5.18 |
| 1435655_at | similar to ribosomal protein L12; predicted gene 7117; small nucleolar RNA, H/ACA box 65; predicted gene 11425; predicted gene 6285; predicted gene 5962; predicted gene 9396; similar to 60S ribosomal protein L12; ribosomal protein L12; predicted gene 6336 | Rpl12 | 15.23 |

**Supplementary Table 4.** Cluster C probe sets, their corresponding gene names/symbols and expression fold changes between liver progenitor cells (LPC)/developed liver (DL). Probe sets are grouped by pathway in order of statistical significance, as determined by Database for Annotation, Visualization and Integrated Discovery and KEGG pathway analysis.

| **Cell Cycle** | **Gene Name** | **Gene Symbol** | **Fold-change (LPC/DL)** |
| --- | --- | --- | --- |
| 1418334_at | DBF4 homolog (S. cerevisiae) | Dbf4 | 43.55 |
| 1437033_a_at, 1436000_a_at, 1418969_at, 1460247_a_at | [S-phase kinase-associated protein 2 (p45)](http://david.abcc.ncifcrf.gov/geneReportFull.jsp?rowids=433925) | Skp2 | 16.36, 49.85, 37.52, 70.57 |
| 1449171_at | [Ttk protein kinase](http://david.abcc.ncifcrf.gov/geneReportFull.jsp?rowids=460501) | Ttk | 42.13 |
| 1423930_at | [anaphase promoting complex subunit 4](http://david.abcc.ncifcrf.gov/geneReportFull.jsp?rowids=466633) | Anapc4 | 8.17 |
| 1427197_at | [ataxia telangiectasia and Rad3 related](http://david.abcc.ncifcrf.gov/geneReportFull.jsp?rowids=450962) | Atr | 3.17 |
| 1424046_at | [budding uninhibited by benzimidazoles 1 homolog (S. cerevisiae)](http://david.abcc.ncifcrf.gov/geneReportFull.jsp?rowids=429759) | Bub1 | 71.05 |
| 1416961_at | [budding uninhibited by benzimidazoles 1 homolog, beta (S. cerevisiae)](http://david.abcc.ncifcrf.gov/geneReportFull.jsp?rowids=433695) | Bub1b | 34.85 |
| 1448314_at | [cell division cycle 2 homolog A (S. pombe)](http://david.abcc.ncifcrf.gov/geneReportFull.jsp?rowids=456008) | Cdk1 | 31.91 |
| 1416664_at | [cell division cycle 20 homolog (S. cerevisiae)](http://david.abcc.ncifcrf.gov/geneReportFull.jsp?rowids=454123) | Cdc20 | 16.46 |
| 1417132_at, 1417131_at | [cell division cycle 25 homolog A (S. pombe)](http://david.abcc.ncifcrf.gov/geneReportFull.jsp?rowids=457114) | Cdc25a | 10.42, 15.48 |
| 1417019_a_at | cell division cycle 6 homolog (S. cerevisiae); predicted gene 9430; similar to cell division cycle 6 homolog | Cdc6 | 9.49 |
| 1426002_a_at | cell division cycle 7 (S. cerevisiae) | Cdc7 | 43.76 |
| 1450677_at | checkpoint kinase 1 homolog (S. pombe) | Chek1 | 86.04 |
| 1417911_at, 1417910_at | cyclin A2 | Ccna2 | 20.38, 10.51 |
| 1450920_at | cyclin B2 | Ccnb2 | 23.74 |
| 1424638_at | cyclin-dependent kinase inhibitor 1A (P21) | Cdkn1a | 35.28 |
| 1449519_at | growth arrest and DNA-damage-inducible 45 alpha | Gadd45a | 7.18 |
| 1450971_at, 1449773_s_at | growth arrest and DNA-damage-inducible 45 beta | Gadd45b | 13.44, 7.82 |
| 1448777_at, 1434079_s_at | minichromosome maintenance deficient 2 mitotin (S. cerevisiae) | Mcm2 | 8.53, 9.80 |
| 1415945_at | minichromosome maintenance deficient 5, cell division cycle 46 (S. cerevisiae) | Mcm5 | 7.76 |
| 1416251_at | minichromosome maintenance deficient 6 (MIS5 homolog, S. pombe) (S. cerevisiae) | Mcm6 | 17.84 |
| 1439269_x_at | minichromosome maintenance deficient 7 (S. cerevisiae) | Mcm7 | 16.69 |
| 1424942_a_at | myelocytomatosis oncogene | Myc | 19.67 |
| 1418226_at, 1418225_at | origin recognition complex, subunit 2-like (S. cerevisiae) | Orc2 | 6.59, 9.84 |
| 1417037_at | origin recognition complex, subunit 6-like (S. cerevisiae) | Orc6 | 14.25 |
| 1448191_at | polo-like kinase 1 (Drosophila) | Plk1 | 26.33 |
| 1419943_s_at, 1448205_at, 1416076_at | predicted gene 8416; predicted gene 5593; cyclin B1; similar to cyclin B1; predicted gene 4870 | Ccnb1 /// Gm5593 /// LOC101056596 | 35.60, 38.80, 38.64 |
| 1424156_at | retinoblastoma-like 1 (p107) | Rbl1 | 10.43 |
| 1421939_a_at | similar to Stromal antigen 1 | Stag1 | 1.62 |
|  |  |  |  |
| **DNA replication** | **Gene Name** | **Gene Symbol** | **Fold-change (LPC/DL)** |
| 1449061_a_at | DNA primase, p49 subunit | Prim1 | 17.28 |
| 1418036_at, 1418035_a_at | DNA primase, p58 subunit | Prim2 | 27.22, 43.47 |
| 1421731_a_at, 1436454_x_at | flap structure specific endonuclease 1 | Fen1 | 12.35, 10.50 |
| 1416641_at | ligase I, DNA, ATP-dependent | Lig1 | 11.46 |
| 1448777_at, 1434079_s_at | minichromosome maintenance deficient 2 mitotin (S. cerevisiae) | Mcm2 | 8.53, 9.80 |
| 1415945_at | minichromosome maintenance deficient 5, cell division cycle 46 (S. cerevisiae) | Mcm5 | 7.76 |
| 1416251_at | minichromosome maintenance deficient 6 (MIS5 homolog, S. pombe) (S. cerevisiae) | Mcm6 | 17.84 |
| 1439269_x_at | minichromosome maintenance deficient 7 (S. cerevisiae) | Mcm7 | 16.69 |
| 1419397_at | polymerase (DNA directed), alpha 1 | Pola1 | 13.53 |
| 1448369_at | polymerase (DNA directed), alpha 2 | Pola2 | 17.52 |
| 1456055_x_at, 1448187_at | polymerase (DNA directed), delta 1, catalytic subunit | Pold1 | 15.17, 13.58 |
| 1448650_a_at | polymerase (DNA directed), epsilon | Pole | 33.73 |
| 1417503_at | replication factor C (activator 1) 2 | Rfc2 | 18.80 |
| 1438161_s_at | replication factor C (activator 1) 4 | Rfc4 | 16.04 |
| 1416433_at | replication protein A2 | Rpa2 | 7.23 |
|  |  |  |  |
| **Pyrimidine metabolism** | **Gene Name** | **Gene Symbol** | **Fold-change (LPC/DL)** |
| 1449061_a_at | DNA primase, p49 subunit | Prim1 | 17.28 |
| 1418036_at, 1418035_a_at | DNA primase, p58 subunit | Prim2 | 27.22, 43.47 |
| 1452830_s_at | carbamoyl-phosphate synthetase 2, aspartate transcarbamylase, and dihydroorotase | Cad | 11.80 |
| 1416563_at | cytidine 5'-triphosphate synthase | Ctps | 74.49 |
| 1419397_at | polymerase (DNA directed), alpha 1 | Pola1 | 13.53 |
| 1448369_at | polymerase (DNA directed), alpha 2 | Pola2 | 17.52 |
| 1456055_x_at, 1448187_at | polymerase (DNA directed), delta 1, catalytic subunit | Pold1 | 15.17, 13.58 |
| 1448650_a_at | polymerase (DNA directed), epsilon | Pole | 33.73 |
| 1416126_at | polymerase (RNA) I polypeptide B | Polr1b | 4.00 |
| 1435057_x_at | polymerase (RNA) I polypeptide E | Polr1e | 6.99 |
| 1449155_at | polymerase (RNA) III (DNA directed) polypeptide G | Polr3g | 3.09 |
| 1416258_at | predicted gene 6091; thymidine kinase 1 | Tk1 | 3.84 |
| 1424991_s_at | thymidylate synthase | Tyms /// Tyms-ps | 13.52 |
| 1455832_a_at | uridine monophosphate synthetase | Umps | 2.77 |
| 1448604_at | uridine-cytidine kinase 2 | Uck2 | 41.96 |
|  |  |  |  |
| **p53 signaling pathway** | **Gene Name** | **Gene Symbol** | **Fold-change (LPC/DL)** |
| 1434279_at | Fas (TNF receptor superfamily member 6) | --- | 5.21 |
| 1452870_at | apoptotic peptidase activating factor 1 | Apaf1 | 18.29 |
| 1427197_at | ataxia telangiectasia and Rad3 related | Atr | 3.17 |
| 1448314_at | cell division cycle 2 homolog A (S. pombe) | Cdk1 | 31.91 |
| 1450677_at | checkpoint kinase 1 homolog (S. pombe) | Chek1 | 86.04 |
| 1450920_at | cyclin B2 | Ccnb2 | 23.74 |
| 1424638_at | cyclin-dependent kinase inhibitor 1A (P21) | Cdkn1a | 35.28 |
| 1449519_at | growth arrest and DNA-damage-inducible 45 alpha | Gadd45a | 7.18 |
| 1449773_s_at, 1450971_at | growth arrest and DNA-damage-inducible 45 beta | Gadd45b | 7.82, 13.44 |
| 1418203_at | phorbol-12-myristate-13-acetate-induced protein 1 | Pmaip1 | 1058.50 |
| 1416076_at, 1419943_s_at, 1448205_at | predicted gene 8416; predicted gene 5593; cyclin B1; similar to cyclin B1; predicted gene 4870 | Ccnb1 /// Gm5593 /// LOC101056596 | 38.64, 35.60, 38.80 |
| 1449353_at | zinc finger matrin type 3 | Zmat3 | 18.48 |
|  |  |  |  |
| **Purine metabolism** | **Gene Name** | **Gene Symbol** | **Fold-change (LPC/DL)** |
| 1449061_a_at | DNA primase, p49 subunit | Prim1 | 17.28 |
| 1418036_at, 1418035_a_at | DNA primase, p58 subunit | Prim2 | 27.22, 43.47 |
| 1418372_at | adenylosuccinate lyase | Adsl | 25.32 |
| 1416843_at | phosphodiesterase 6D, cGMP-specific, rod, delta | Pde6d | 6.34 |
| 1420637_at | phosphoribosyl pyrophosphate synthetase 2 | Prps2 | 6.39 |
| 1419397_at | polymerase (DNA directed), alpha 1 | Pola1 | 13.53 |
| 1448369_at | polymerase (DNA directed), alpha 2 | Pola2 | 17.52 |
| 1456055_x_at, 1448187_at | polymerase (DNA directed), delta 1, catalytic subunit | Pold1 | 15.17, 13.58 |
| 1448650_a_at | polymerase (DNA directed), epsilon | Pole | 33.73 |
| 1416126_at | polymerase (RNA) I polypeptide B | Polr1b | 4.00 |
| 1435057_x_at | polymerase (RNA) I polypeptide E | Polr1e | 6.99 |
| 1449155_at | polymerase (RNA) III (DNA directed) polypeptide G | Polr3g | 3.09 |
|  |  |  |  |
| **Oocyte Meiosis** | **Gene Name** | **Gene Symbol** | **Fold-change (LPC/DL)** |
| 1423930_at | anaphase promoting complex subunit 4 | Anapc4 | 8.17 |
| 1424511_at | aurora kinase A | Aurka | 17.57 |
| 1424046_at | budding uninhibited by benzimidazoles 1 homolog (S. cerevisiae) | Bub1 | 71.05 |
| 1448314_at | cell division cycle 2 homolog A (S. pombe) | Cdk1 | 31.91 |
| 1416664_at | cell division cycle 20 homolog (S. cerevisiae) | Cdc20 | 16.46 |
| 1450920_at | cyclin B2 | Ccnb2 | 23.74 |
| 1417297_at | inositol 1,4,5-triphosphate receptor 3 | Itpr3 | Not expressed in DL |
| 1448191_at | polo-like kinase 1 (Drosophila) | Plk1 | 26.33 |
| 1416076_at, 1419943_s_at, 1448205_at | predicted gene 8416; predicted gene 5593; cyclin B1; similar to cyclin B1; predicted gene 4870 | Ccnb1 /// Gm5593 /// LOC101056596 | 38.64, 35.60, 38.80 |
| 1438174_x_at | protein phosphatase 2 (formerly 2A), regulatory subunit A (PR 65), alpha isoform | Ppp2r1a | 10.45 |

**Supplementary Table 5.** Cluster D probe sets, their corresponding gene names/symbols and expression fold changes between liver progenitor cells (LPC)/developed liver (DL). Probe sets are grouped by pathway in order of statistical significance, as determined by Database for Annotation, Visualization and Integrated Discovery and KEGG pathway analysis.

| **Complement and coagulation cascades** | **Gene Name** | **Gene Symbol** | **Fold-change (LPC/DL)** |
| --- | --- | --- | --- |
| 1418897_at | coagulation factor II | F2 | 9.77E-04 |
| 1427393_at | coagulation factor IX | F9 | Not expressed in LPCs |
| 1449269_at, 1418907_at | coagulation factor V; similar to Murine coagulation factor V | F5 | 1.84E-03,  1.61E-03 |
| 1419321_at | coagulation factor VII | F7 | Not expressed in LPCs |
| 1449305_at, 1418993_s_at, 1418992_at | coagulation factor X | F10 | Not expressed in LPCs, Not expressed in LPCs, Not expressed in LPCs |
| 1451788_at | coagulation factor XI | F11 | Not expressed in LPCs |
| 1420496_at | coagulation factor XII (Hageman factor) | F12 | Not expressed in LPCs |
| 1419131_at | coagulation factor XIII, beta subunit | F13b | Not expressed in LPCs |
| 1449401_at | complement component 1, q subcomponent, C chain | C1qc | Not expressed in LPCs |
| 1417381_at | complement component 1, q subcomponent, alpha polypeptide | C1qa | Not expressed in LPCs |
| 1437726_x_at, 1434366_x_at, 1417063_at | complement component 1, q subcomponent, beta polypeptide | C1qb | Not expressed in LPCs, Not expressed in LPCs, Not expressed in LPCs |
| 1449308_at | complement component 6 | C6 | Not expressed in LPCs |
| 1428012_at | complement component 8, alpha polypeptide | C8a | Not expressed in LPCs |
| 1427472_a_at | complement component 8, beta polypeptide | C8b | Not expressed in LPCs |
| 1416025_at | fibrinogen gamma chain | Fgg | Not expressed in LPCs |
| 1419407_at | hemolytic complement | Hc | Not expressed in LPCs |
| 1449034_at | kallikrein B, plasma 1 | Klkb1 | Not expressed in LPCs |
| 1426045_at | kininogen 1 | Kng1 | Not expressed in LPCs |
| 1419677_at | mannan-binding lectin serine peptidase 1 | Masp1 | Not expressed in LPCs |
| 1451759_at, 1420524_a_at | mannan-binding lectin serine peptidase 2 | Masp2 | Not expressed in LPCs, Not expressed in LPCs |
| 1419578_at | mannose-binding lectin (protein A) 1 | Mbl1 | Not expressed in LPCs |
| 1418787_at | mannose-binding lectin (protein C) 2 | Mbl2 | Not expressed in LPCs |
| 1416729_at | plasminogen | Plg | Not expressed in LPCs |
| 1417909_at | serine (or cysteine) peptidase inhibitor, clade C (antithrombin), member 1 | Serpinc1 | 2.74E-04 |
| 1417498_at | serine (or cysteine) peptidase inhibitor, clade F, member 2 | Serpinf2 | Not expressed in LPCs |
|  |  |  |  |
| **Drug metabolism (cytochrome P450)** | **Gene Name** | **Gene Symbol** | **Fold-change (LPC/DL)** |
| 1450133_at | UDP glucuronosyltransferase 2 family, polypeptide A3 | Ugt2a3 | Not expressed in LPCs |
| 1424934_at | UDP glucuronosyltransferase 2 family, polypeptide B1 | Ugt2b1 | Not expressed in LPCs |
| 1422070_at | alcohol dehydrogenase 4 (class II), pi polypeptide | Adh4 | Not expressed in LPCs |
| 1450715_at | cytochrome P450, family 1, subfamily a, polypeptide 2 | Cyp1a2 | Not expressed in LPCs |
| 1418821_at | cytochrome P450, family 2, subfamily a, polypeptide 12; cytochrome P450, family 2, subfamily a, polypeptide 22 | Cyp2a12 | Not expressed in LPCs |
| 1425645_s_at | cytochrome P450, family 2, subfamily b, polypeptide 10 | Cyp2b10 | Not expressed in LPCs |
| 1418653_at | cytochrome P450, family 2, subfamily c, polypeptide 50 | Cyp2c50 | Not expressed in LPCs |
| 1424273_at | cytochrome P450, family 2, subfamily c, polypeptide 70 | Cyp2c70 | Not expressed in LPCs |
| 1418113_at | cytochrome P450, family 2, subfamily d, polypeptide 10 | Cyp2d10 | Not expressed in LPCs |
| 1448683_at | cytochrome P450, family 2, subfamily d, polypeptide 26 | Cyp2d26 | Not expressed in LPCs |
| 1419349_a_at | cytochrome P450, family 2, subfamily d, polypeptide 9 | Cyp2d9 | Not expressed in LPCs |
| 1415994_at | cytochrome P450, family 2, subfamily e, polypeptide 1 | Cyp2e1 | Not expressed in LPCs |
| 1416809_at | cytochrome P450, family 3, subfamily a, polypeptide 11 | Cyp3a11 | Not expressed in LPCs |
| 1419704_at | cytochrome P450, family 3, subfamily a, polypeptide 41A; cytochrome P450, family 3, subfamily a, polypeptide 41B | Cyp3a41a /// Cyp3a41b | Not expressed in LPCs |
| 1427474_s_at, 1427473_at | glutathione S-transferase, mu 3 | Gstm3 | 5.97E-03, Not expressed in LPCs |
| 1417883_at | glutathione S-transferase, theta 2 | Gstt2 | 5.89E-03 |
| 1419094_at | similar to cytochrome P450; CYP2C37; cytochrome P450, family 2. subfamily c, polypeptide 37 | Cyp2c37 /// LOC101056460 | Not expressed in LPCs |
| 1455457_at | similar to cytochrome P450; cytochrome P450, family 2, subfamily c, polypeptide 54 | Cyp2c54 | 9.21E-04 |
|  |  |  |  |
| **Retinol metabolism** | **Gene Name** | **Gene Symbol** | **Fold-change (LPC/DL)** |
| 1450133_at | UDP glucuronosyltransferase 2 family, polypeptide A3 | Ugt2a3 | Not expressed in LPCs |
| 1424934_at | UDP glucuronosyltransferase 2 family, polypeptide B1 | Ugt2b1 | Not expressed in LPCs |
| 1422070_at | alcohol dehydrogenase 4 (class II), pi polypeptide | Adh4 | Not expressed in LPCs |
| 1450715_at | cytochrome P450, family 1, subfamily a, polypeptide 2 | Cyp1a2 | Not expressed in LPCs |
| 1418821_at | cytochrome P450, family 2, subfamily a, polypeptide 12; cytochrome P450, family 2, subfamily a, polypeptide 22 | Cyp2a12 | Not expressed in LPCs |
| 1425645_s_at | cytochrome P450, family 2, subfamily b, polypeptide 10 | Cyp2b10 | Not expressed in LPCs |
| 1418653_at | cytochrome P450, family 2, subfamily c, polypeptide 50 | Cyp2c50 | Not expressed in LPCs |
| 1424273_at | cytochrome P450, family 2, subfamily c, polypeptide 70 | Cyp2c70 | Not expressed in LPCs |
| 1419430_at | cytochrome P450, family 26, subfamily a, polypeptide 1 | Cyp26a1 | Not expressed in LPCs |
| 1416809_at | cytochrome P450, family 3, subfamily a, polypeptide 11 | Cyp3a11 | Not expressed in LPCs |
| 1419704_at | cytochrome P450, family 3, subfamily a, polypeptide 41A; cytochrome P450, family 3, subfamily a, polypeptide 41B | Cyp3a41a /// Cyp3a41b | Not expressed in LPCs |
| 1424352_at | cytochrome P450, family 4, subfamily a, polypeptide 12a | Cyp4a12a | 1.50E-03 |
| 1423257_at | cytochrome P450, family 4, subfamily a, polypeptide 14 | Cyp4a14 | Not expressed in LPCs |
| 1420541_at | retinol dehydrogenase 16 | Rdh16 | Not expressed in LPCs |
| 1424853_s_at | similar to DNA-directed RNA polymerase II 7.6 kDa polypeptide (RPB10) (RPB7.6) (RPABC5); hypothetical protein LOC100044218; predicted gene 13015; polymerase (RNA) II (DNA directed) polypeptide L; cytochrome P450, family 4, subfamily a, polypeptide 31; cytochrome P450, family 4, subfamily a, polypeptide 32; predicted gene 10774; cytochrome P450, family 4, subfamily a, polypeptide 10 | Cyp4a10 /// Cyp4a31 | 5.08E-04 |
| 1419094_at | similar to cytochrome P450; CYP2C37; cytochrome P450, family 2. subfamily c, polypeptide 37 | Cyp2c37 /// LOC101056460 | Not expressed in LPCs |
| 1455457_at | similar to cytochrome P450; cytochrome P450, family 2, subfamily c, polypeptide 54 | Cyp2c54 | 9.21E-04 |
|  |  |  |  |
| **Drug metabolism (other enzymes)** | **Gene Name** | **Gene Symbol** | **Fold-change (LPC/DL)** |
| 1450133_at | UDP glucuronosyltransferase 2 family, polypeptide A3 | Ugt2a3 | Not expressed in LPCs |
| 1424934_at | UDP glucuronosyltransferase 2 family, polypeptide B1 | Ugt2b1 | Not expressed in LPCs |
| 1449081_at, 1435371_x_at, 1435370_a_at | carboxylesterase 3 | Ces1d | Not expressed in LPCs, Not expressed in LPCs, 1.82E-03 |
| 1427137_at | carboxylesterase 5 | Ces2e | 7.48E-03 |
| 1418821_at | cytochrome P450, family 2, subfamily a, polypeptide 12; cytochrome P450, family 2, subfamily a, polypeptide 22 | Cyp2a12 | Not expressed in LPCs |
| 1416809_at | cytochrome P450, family 3, subfamily a, polypeptide 11 | Cyp3a11 | Not expressed in LPCs |
| 1419704_at | cytochrome P450, family 3, subfamily a, polypeptide 41A; cytochrome P450, family 3, subfamily a, polypeptide 41B | Cyp3a41a /// Cyp3a41b | Not expressed in LPCs |
| 1425688_a_at, 1436291_a_at | dihydropyrimidinase | Dpys | Not expressed in LPCs, Not expressed in LPCs |
| 1427945_at, 1427946_s_at | dihydropyrimidine dehydrogenase | Dpyd | Not expressed in LPCs, 8.61E-04 |
| 1416913_at | esterase 1 | Ces1c | Not expressed in LPCs |
| 1419510_at | esterase 22 | Ces1e | Not expressed in LPCs |
| 1451600_s_at | esterase 31; predicted gene 4738 | Ces3a /// Ces3b | Not expressed in LPCs |
| 1460244_at | ureidopropionase, beta | Upb1 | 3.11E-03 |
| 1451548_at, 1424969_s_at | uridine phosphorylase 2 | Upp2 | Not expressed in LPCs, Not expressed in LPCs |
|  |  |  |  |
| **Metabolism of xenobiotics by cytochrome P450** | **Gene Name** | **Gene Symbol** | **Fold-change (LPC/DL)** |
| 1450133_at | UDP glucuronosyltransferase 2 family, polypeptide A3 | Ugt2a3 | Not expressed in LPCs |
| 1424934_at | UDP glucuronosyltransferase 2 family, polypeptide B1 | Ugt2b1 | Not expressed in LPCs |
| 1422070_at | alcohol dehydrogenase 4 (class II), pi polypeptide | Adh4 | Not expressed in LPCs |
| 1450715_at | cytochrome P450, family 1, subfamily a, polypeptide 2 | Cyp1a2 | Not expressed in LPCs |
| 1425645_s_at | cytochrome P450, family 2, subfamily b, polypeptide 10 | Cyp2b10 | Not expressed in LPCs |
| 1418653_at | cytochrome P450, family 2, subfamily c, polypeptide 50 | Cyp2c50 | Not expressed in LPCs |
| 1424273_at | cytochrome P450, family 2, subfamily c, polypeptide 70 | Cyp2c70 | Not expressed in LPCs |
| 1415994_at | cytochrome P450, family 2, subfamily e, polypeptide 1 | Cyp2e1 | Not expressed in LPCs |
| 1448792_a_at | cytochrome P450, family 2, subfamily f, polypeptide 2 | Cyp2f2 | Not expressed in LPCs |
| 1416809_at | cytochrome P450, family 3, subfamily a, polypeptide 11 | Cyp3a11 | Not expressed in LPCs |
| 1419704_at | cytochrome P450, family 3, subfamily a, polypeptide 41A; cytochrome P450, family 3, subfamily a, polypeptide 41B | Cyp3a41a /// Cyp3a41b | Not expressed in LPCs |
| 1427474_s_at, 1427473_at | glutathione S-transferase, mu 3 | Gtsm3 | 5.97E-03, Not expressed in LPCs |
| 1417883_at | glutathione S-transferase, theta 2 | Gstt2 | 5.89E-03 |
| 1419094_at | similar to cytochrome P450; CYP2C37; cytochrome P450, family 2. subfamily c, polypeptide 37 | Cyp2c37 /// LOC101056460 | Not expressed in LPCs |
| 1455457_at | similar to cytochrome P450; cytochrome P450, family 2, subfamily c, polypeptide 54 | Cyp2c54 | 9.21E-04 |
|  |  |  |  |
| **Glycine, serine and threonine metabolism** | **Gene Name** | **Gene Symbol** | **Fold-change (LPC/DL)** |
| 1418833_at | alanine-glyoxylate aminotransferase | Agxt | Not expressed in LPCs |
| 1451675_a_at | aminolevulinic acid synthase 2, erythroid | Alas2 | Not expressed in LPCs |
| 1450624_at | betaine-homocysteine methyltransferase | Bhmt | Not expressed in LPCs |
| 1455435_s_at | choline dehydrogenase | Chdh | 1.03E-02 |
| 1425623_a_at | cystathionine beta-synthase | Cbs | 6.62E-03 |
| 1452311_at | dimethylglycine dehydrogenase precursor | Dmgdh | Not expressed in LPCs |
| 1424994_at | glycerate kinase | Glyctk | 1.86E-02 |
| 1417422_at | glycine N-methyltransferase | Gnmt | Not expressed in LPCs |
| 1448426_at | sarcosine dehydrogenase | Sardh | 4.41E-03 |
| 1424744_at | serine dehydratase | Sds | Not expressed in LPCs |
| 1438550_x_at | serine racemase | Srr | 3.16E-02 |
|  |  |  |  |
| **PPAR signaling pathway** | **Gene Name** | **Gene Symbol** | **Fold-change (LPC/DL)** |
| 1423858_a_at | 3-hydroxy-3-methylglutaryl-Coenzyme A synthase 2 | Hmgcs2 | 7.69E-04 |
| 1423166_at | CD36 antigen | Cd36 | 1.62E-02 |
| 1419233_x_at, 1419232_a_at, 1455201_x_at, 1438840_x_at | apolipoprotein A-I | Apoa1 | Not expressed in LPCs, 2.52E-04,  6.24E-04,  5.40E-04 |
| 1417950_a_at | apolipoprotein A-II | Apoa2 | Not expressed in LPCs |
| 1417610_at, 1419857_at | apolipoprotein A-V | Apoa5 | Not expressed in LPCs, Not expressed in LPCs |
| 1418278_at | apolipoprotein C-III | Apoc3 | Not expressed in LPCs |
| 1424352_at | cytochrome P450, family 4, subfamily a, polypeptide 12a | Cyp4a12a | 1.50E-03 |
| 1423257_at | cytochrome P450, family 4, subfamily a, polypeptide 14 | Cyp4a14 | Not expressed in LPCs |
| 1438743_at | cytochrome P450, family 7, subfamily a, polypeptide 1 | Cyp7a1 | 2.21E-03 |
| 1449309_at | cytochrome P450, family 8, subfamily b, polypeptide 1 | Cyp8b1 | 1.88E-03 |
| 1449051_at | peroxisome proliferator activated receptor alpha | Ppara | Not expressed in LPCs |
| 1423439_at | phosphoenolpyruvate carboxykinase 1, cytosolic | Pck1 | Not expressed in LPCs |
| 1424853_s_at | similar to DNA-directed RNA polymerase II 7.6 kDa polypeptide (RPB10) (RPB7.6) (RPABC5); hypothetical protein LOC100044218; predicted gene 13015; polymerase (RNA) II (DNA directed) polypeptide L; cytochrome P450, family 4, subfamily a, polypeptide 31; cytochrome P450, family 4, subfamily a, polypeptide 32; predicted gene 10774; cytochrome P450, family 4, subfamily a, polypeptide 10 | Cyp4a10 /// Cyp4a31 | 5.08E-04 |
| 1416316_at | solute carrier family 27 (fatty acid transporter), member 2 | Slc27a2 | Not expressed in LPCs |
| 1449112_at | solute carrier family 27 (fatty acid transporter), member 5 | Slc27a5 | Not expressed in LPCs |
|  |  |  |  |
| **Linoleic acid metabolism** | **Gene Name** | **Gene Symbol** | **Fold-change (LPC/DL)** |
| 1417085_at | aldo-keto reductase family 1, member C6 | Akr1c6 | Not expressed in LPCs |
| 1450715_at | cytochrome P450, family 1, subfamily a, polypeptide 2 | Cyp1a2 | Not expressed in LPCs |
| 1418653_at | cytochrome P450, family 2, subfamily c, polypeptide 50 | Cyp2c50 | Not expressed in LPCs |
| 1424273_at | cytochrome P450, family 2, subfamily c, polypeptide 70 | Cyp2c70 | Not expressed in LPCs |
| 1415994_at | cytochrome P450, family 2, subfamily e, polypeptide 1 | Cyp2e1 | Not expressed in LPCs |
| 1417532_at, 1417531_at | cytochrome P450, family 2, subfamily j, polypeptide 5 | Cyp2j5 | Not expressed in LPCs, Not expressed in LPCs |
| 1416809_at | cytochrome P450, family 3, subfamily a, polypeptide 11 | Cyp3a11 | Not expressed in LPCs |
| 1419704_at | cytochrome P450, family 3, subfamily a, polypeptide 41A; cytochrome P450, family 3, subfamily a, polypeptide 41B | Cyp3a41a /// Cyp3a41b | Not expressed in LPCs |
| 1419614_at | phospholipase A2, group XIIB | Pla2g12b | 5.99E-03 |
| 1419094_at | similar to cytochrome P450; CYP2C37; cytochrome P450, family 2. subfamily c, polypeptide 37 | Cyp2c37 /// LOC101056460 | Not expressed in LPCs |
| 1455457_at | similar to cytochrome P450; cytochrome P450, family 2, subfamily c, polypeptide 54 | Cyp2c54 | 9.21E-04 |
|  |  |  |  |
| **Arachidonic acid metabolism** | **Gene Name** | **Gene Symbol** | **Fold-change (LPC/DL)** |
| 1425645_s_at | cytochrome P450, family 2, subfamily b, polypeptide 10 | Cyp2b10 | Not expressed in LPCs |
| 1418653_at | cytochrome P450, family 2, subfamily c, polypeptide 50 | Cyp2c50 | Not expressed in LPCs |
| 1424273_at | cytochrome P450, family 2, subfamily c, polypeptide 70 | Cyp2c70 | Not expressed in LPCs |
| 1415994_at | cytochrome P450, family 2, subfamily e, polypeptide 1 | Cyp2e1 | Not expressed in LPCs |
| 1417532_at, 1417531_at | cytochrome P450, family 2, subfamily j, polypeptide 5 | Cyp2j5 | Not expressed in LPCs, Not expressed in LPCs |
| 1424352_at | cytochrome P450, family 4, subfamily a, polypeptide 12a | Cyp4a12a | 1.50E-03 |
| 1423257_at | cytochrome P450, family 4, subfamily a, polypeptide 14 | Cyp4a14 | Not expressed in LPCs |
| 1419559_at | cytochrome P450, family 4, subfamily f, polypeptide 14 | Cyp4f14 | Not expressed in LPCs |
| 1449316_at | cytochrome P450, family 4, subfamily f, polypeptide 15 | Cyp4f15 | Not expressed in LPCs |
| 1419614_at | phospholipase A2, group XIIB | Pla2g12b | 5.99E-03 |
| 1424853_s_at | similar to DNA-directed RNA polymerase II 7.6 kDa polypeptide (RPB10) (RPB7.6) (RPABC5); hypothetical protein LOC100044218; predicted gene 13015; polymerase (RNA) II (DNA directed) polypeptide L; cytochrome P450, family 4, subfamily a, polypeptide 31; cytochrome P450, family 4, subfamily a, polypeptide 32; predicted gene 10774; cytochrome P450, family 4, subfamily a, polypeptide 10 | Cyp4a10 /// Cyp4a31 | 5.08E-04 |
| 1419094_at | similar to cytochrome P450; CYP2C37; cytochrome P450, family 2. subfamily c, polypeptide 37 | Cyp2c37 /// LOC101056460 | Not expressed in LPCs |
| 1455457_at | similar to cytochrome P450; cytochrome P450, family 2, subfamily c, polypeptide 54 | Cyp2c54 | 9.21E-04 |

**Supplementary Table 6.** Summary of the promoter elements overrepresented in the genes from cluster A, cluster C and cluster D.

| **Gene\Factor** | NRF-2 | USF | CRE-BP1/ c-Jun | CREB | Gfi-1 | E2F | MIF-1 | COMP1 | AP-1 | AREB6 | CP2 | MyoD | Nkx2-5 | HNF-1 | HNF-4 | NFκB | COUP-TF/ HNF-4 | Retroviral Poly A | Brn-2 |
| --- | --- | --- | --- | --- | --- | --- | --- | --- | --- | --- | --- | --- | --- | --- | --- | --- | --- | --- | --- |
|  |  |  |  |  |  |  |  |  |  |  |  |  |  |  |  |  |  |  |  |
| NM_009072 | 1 | 0 | 0 | 0 | 0 | 0 | 0 | 0 | 0 | 0 | 0 | 0 | 0 | 0 | 0 | 0 | 0 | 0 | 0 |
| NM_007581 | 1 | 0 | 0 | 0 | 0 | 0 | 0 | 0 | 0 | 0 | 0 | 0 | 0 | 0 | 0 | 0 | 0 | 0 | 0 |
| NM_007396 | 1 | 0 | 0 | 0 | 0 | 0 | 0 | 0 | 0 | 0 | 1 | 0 | 0 | 0 | 0 | 0 | 0 | 0 | 0 |
| NM_008513 | 1 | 1 | 0 | 0 | 0 | 0 | 0 | 0 | 0 | 0 | 0 | 0 | 1 | 0 | 0 | 0 | 0 | 0 | 0 |
| NM_011348 | 0 | 1 | 0 | 0 | 0 | 0 | 0 | 0 | 0 | 0 | 0 | 0 | 0 | 0 | 0 | 0 | 0 | 0 | 0 |
| NM_009829 | 0 | 1 | 0 | 0 | 0 | 0 | 0 | 0 | 0 | 0 | 0 | 1 | 0 | 0 | 0 | 0 | 0 | 0 | 0 |
| NM_026529 | 0 | 2 | 0 | 0 | 0 | 0 | 0 | 0 | 0 | 0 | 0 | 0 | 0 | 1 | 0 | 0 | 0 | 0 | 0 |
| NM_053268 | 0 | 1 | 0 | 0 | 0 | 0 | 0 | 1 | 0 | 0 | 0 | 0 | 0 | 0 | 0 | 0 | 0 | 0 | 0 |
| NM_009830 | 0 | 2 | 0 | 0 | 0 | 0 | 0 | 0 | 0 | 0 | 0 | 0 | 0 | 0 | 1 | 0 | 0 | 0 | 0 |
| NM_001025365 | 0 | 1 | 0 | 0 | 0 | 0 | 0 | 0 | 0 | 0 | 0 | 0 | 0 | 0 | 1 | 0 | 0 | 0 | 0 |
| NM_023813 | 0 | 1 | 2 | 2 | 0 | 0 | 0 | 0 | 0 | 0 | 0 | 0 | 0 | 0 | 1 | 0 | 0 | 0 | 0 |
| NM_001025438 | 0 | 1 | 2 | 2 | 0 | 0 | 0 | 0 | 0 | 0 | 0 | 0 | 0 | 0 | 1 | 0 | 0 | 0 | 0 |
| NM_001025439 | 0 | 1 | 2 | 2 | 0 | 0 | 0 | 0 | 0 | 0 | 0 | 0 | 0 | 0 | 1 | 0 | 0 | 0 | 0 |
| NM_026255 | 0 | 0 | 2 | 2 | 0 | 0 | 0 | 1 | 0 | 0 | 0 | 0 | 0 | 0 | 0 | 0 | 0 | 0 | 0 |
| NM_206958 | 0 | 0 | 2 | 2 | 0 | 0 | 0 | 0 | 0 | 0 | 0 | 0 | 0 | 0 | 0 | 0 | 0 | 0 | 0 |
| NM_019919 | 0 | 0 | 2 | 2 | 0 | 0 | 0 | 0 | 0 | 0 | 0 | 0 | 0 | 0 | 0 | 0 | 0 | 0 | 0 |
| NM_009704 | 0 | 0 | 2 | 2 | 0 | 0 | 0 | 0 | 0 | 0 | 0 | 0 | 0 | 0 | 0 | 0 | 0 | 0 | 0 |
| NM_010200 | 0 | 0 | 1 | 0 | 0 | 0 | 0 | 1 | 1 | 0 | 0 | 0 | 0 | 0 | 0 | 0 | 0 | 0 | 0 |
| NM_023525 | 0 | 0 | 0 | 0 | 1 | 0 | 0 | 0 | 0 | 0 | 0 | 0 | 0 | 0 | 0 | 0 | 0 | 0 | 0 |
| NM_009517 | 0 | 0 | 0 | 0 | 1 | 0 | 0 | 1 | 0 | 0 | 0 | 0 | 0 | 0 | 0 | 0 | 0 | 0 | 0 |
| NM_001025779 | 0 | 0 | 0 | 0 | 0 | 1 | 0 | 0 | 0 | 0 | 0 | 0 | 0 | 0 | 0 | 0 | 0 | 0 | 0 |
| NM_009387 | 0 | 0 | 0 | 0 | 0 | 1 | 0 | 0 | 0 | 0 | 0 | 0 | 2 | 0 | 0 | 0 | 0 | 0 | 0 |
| NM_007659 | 0 | 0 | 0 | 0 | 0 | 1 | 0 | 0 | 0 | 0 | 0 | 0 | 0 | 0 | 0 | 0 | 0 | 0 | 0 |
| NM_011799 | 0 | 0 | 0 | 0 | 0 | 1 | 0 | 0 | 0 | 0 | 0 | 0 | 0 | 0 | 0 | 0 | 0 | 0 | 0 |
| NM_007658 | 0 | 0 | 0 | 0 | 0 | 1 | 0 | 0 | 0 | 0 | 0 | 0 | 0 | 0 | 0 | 0 | 0 | 0 | 0 |
| NM_026260 | 0 | 0 | 0 | 0 | 0 | 0 | 1 | 0 | 0 | 0 | 0 | 0 | 1 | 0 | 1 | 0 | 0 | 0 | 1 |
| NM_172301 | 0 | 0 | 0 | 0 | 0 | 0 | 1 | 0 | 0 | 0 | 0 | 0 | 0 | 0 | 0 | 0 | 0 | 0 | 0 |
| NM_008892 | 0 | 0 | 1 | 0 | 0 | 1 | 0 | 1 | 0 | 0 | 0 | 0 | 0 | 1 | 0 | 0 | 0 | 0 | 0 |
| NM_007630 | 0 | 0 | 0 | 0 | 0 | 0 | 0 | 1 | 0 | 0 | 0 | 0 | 0 | 0 | 0 | 0 | 0 | 0 | 0 |
| NM_007669 | 0 | 0 | 0 | 0 | 0 | 0 | 0 | 1 | 0 | 0 | 0 | 0 | 0 | 0 | 0 | 0 | 0 | 0 | 0 |
| NM_009086 | 0 | 0 | 0 | 0 | 0 | 0 | 0 | 1 | 0 | 0 | 0 | 0 | 0 | 0 | 0 | 0 | 0 | 0 | 0 |
| NM_008893 | 0 | 0 | 0 | 0 | 0 | 0 | 0 | 1 | 0 | 0 | 0 | 0 | 0 | 1 | 0 | 0 | 0 | 0 | 0 |
| NM_013787 | 0 | 0 | 0 | 0 | 0 | 0 | 0 | 1 | 0 | 0 | 0 | 0 | 0 | 0 | 1 | 0 | 0 | 0 | 0 |
| NM_030724 | 0 | 0 | 0 | 0 | 0 | 0 | 0 | 1 | 0 | 0 | 0 | 0 | 0 | 0 | 0 | 0 | 0 | 0 | 0 |
| NM_009634 | 0 | 0 | 0 | 0 | 0 | 0 | 0 | 1 | 0 | 0 | 0 | 0 | 0 | 0 | 1 | 0 | 0 | 0 | 0 |
| NM_008921 | 0 | 0 | 0 | 0 | 0 | 0 | 0 | 1 | 0 | 0 | 0 | 0 | 1 | 0 | 0 | 0 | 0 | 0 | 0 |
| NM_027411 | 0 | 0 | 0 | 0 | 0 | 0 | 0 | 1 | 0 | 0 | 0 | 0 | 0 | 0 | 1 | 0 | 0 | 0 | 0 |
| NM_013749 | 0 | 1 | 2 | 2 | 0 | 0 | 0 | 1 | 0 | 0 | 0 | 0 | 2 | 0 | 0 | 0 | 0 | 0 | 0 |
| NM_145588 | 1 | 0 | 0 | 0 | 0 | 0 | 0 | 1 | 0 | 0 | 0 | 0 | 1 | 0 | 0 | 0 | 0 | 0 | 0 |
| NM_145468 | 0 | 0 | 0 | 0 | 0 | 0 | 0 | 1 | 0 | 0 | 0 | 0 | 0 | 0 | 1 | 0 | 0 | 0 | 0 |
| NM_026701 | 0 | 0 | 0 | 0 | 0 | 0 | 0 | 0 | 1 | 1 | 0 | 0 | 0 | 0 | 0 | 0 | 0 | 0 | 0 |
| NM_007572 | 0 | 0 | 0 | 0 | 0 | 0 | 0 | 0 | 1 | 0 | 0 | 0 | 0 | 0 | 0 | 0 | 0 | 0 | 0 |
| NM_133995 | 0 | 0 | 0 | 0 | 0 | 0 | 0 | 0 | 1 | 0 | 0 | 0 | 0 | 0 | 0 | 0 | 0 | 0 | 0 |
| NM_028066 | 0 | 0 | 0 | 0 | 0 | 0 | 0 | 0 | 0 | 0 | 1 | 0 | 0 | 0 | 0 | 0 | 0 | 0 | 0 |
| NM_011144 | 0 | 0 | 0 | 0 | 0 | 0 | 0 | 0 | 0 | 0 | 1 | 0 | 0 | 0 | 0 | 0 | 0 | 0 | 0 |
| NM_009247 | 0 | 0 | 0 | 0 | 0 | 0 | 0 | 0 | 0 | 0 | 0 | 2 | 0 | 0 | 0 | 1 | 0 | 0 | 0 |
| NM_009777 | 0 | 0 | 0 | 0 | 0 | 0 | 0 | 0 | 0 | 0 | 0 | 1 | 0 | 0 | 0 | 0 | 0 | 0 | 0 |
| NM_144512 | 0 | 0 | 0 | 0 | 0 | 0 | 0 | 0 | 0 | 0 | 0 | 1 | 1 | 0 | 0 | 0 | 0 | 0 | 0 |
| NM_010776 | 0 | 0 | 0 | 0 | 0 | 0 | 0 | 0 | 0 | 0 | 0 | 0 | 2 | 0 | 0 | 0 | 0 | 0 | 0 |
| NM_146148 | 0 | 0 | 0 | 0 | 0 | 0 | 0 | 1 | 0 | 0 | 0 | 0 | 1 | 0 | 0 | 0 | 0 | 0 | 0 |
| NM_007979 | 0 | 0 | 0 | 0 | 0 | 0 | 0 | 0 | 0 | 0 | 0 | 0 | 1 | 0 | 0 | 0 | 0 | 0 | 0 |
| NM_007574 | 0 | 0 | 0 | 0 | 0 | 0 | 0 | 0 | 0 | 0 | 0 | 0 | 2 | 0 | 0 | 0 | 0 | 0 | 0 |
| NM_008555 | 0 | 1 | 0 | 0 | 0 | 0 | 0 | 0 | 0 | 0 | 0 | 0 | 1 | 0 | 0 | 0 | 0 | 0 | 0 |
| NM_007972 | 0 | 0 | 0 | 0 | 0 | 0 | 0 | 0 | 0 | 0 | 0 | 0 | 1 | 0 | 0 | 0 | 0 | 0 | 0 |
| NM_152811 | 0 | 0 | 0 | 0 | 0 | 0 | 0 | 1 | 0 | 0 | 0 | 0 | 1 | 0 | 0 | 0 | 0 | 0 | 0 |
| NM_010361 | 0 | 1 | 0 | 0 | 0 | 0 | 0 | 2 | 0 | 0 | 0 | 0 | 1 | 0 | 0 | 0 | 0 | 0 | 0 |
| NM_009993 | 0 | 0 | 0 | 0 | 0 | 0 | 0 | 0 | 0 | 0 | 0 | 0 | 1 | 0 | 0 | 0 | 0 | 0 | 0 |
| NM_010011 | 0 | 0 | 0 | 0 | 0 | 0 | 0 | 0 | 0 | 0 | 0 | 0 | 1 | 0 | 0 | 0 | 0 | 0 | 0 |
| NM_133882 | 0 | 0 | 0 | 0 | 0 | 0 | 0 | 1 | 0 | 1 | 0 | 0 | 1 | 0 | 0 | 0 | 0 | 0 | 0 |
| NM_134144 | 0 | 0 | 0 | 0 | 0 | 0 | 0 | 0 | 0 | 1 | 0 | 0 | 1 | 0 | 0 | 0 | 0 | 0 | 0 |
| NM_008877 | 0 | 0 | 0 | 0 | 0 | 0 | 0 | 0 | 0 | 0 | 0 | 0 | 0 | 1 | 0 | 0 | 0 | 0 | 0 |
| NM_007824 | 0 | 0 | 0 | 0 | 0 | 0 | 0 | 0 | 0 | 0 | 0 | 0 | 0 | 1 | 0 | 0 | 0 | 0 | 0 |
| NM_010406 | 0 | 0 | 0 | 0 | 0 | 0 | 0 | 0 | 0 | 0 | 0 | 0 | 0 | 1 | 0 | 0 | 0 | 0 | 0 |
| NM_010007 | 0 | 0 | 0 | 0 | 0 | 0 | 0 | 0 | 0 | 0 | 0 | 0 | 0 | 1 | 0 | 0 | 0 | 0 | 0 |
| NM_007817 | 0 | 0 | 0 | 0 | 0 | 0 | 0 | 0 | 0 | 0 | 0 | 0 | 1 | 1 | 0 | 0 | 0 | 0 | 0 |
| NM_008878 | 0 | 0 | 0 | 0 | 0 | 0 | 0 | 0 | 0 | 0 | 0 | 0 | 1 | 1 | 2 | 0 | 0 | 0 | 0 |
| NM_009888 | 0 | 0 | 0 | 0 | 0 | 0 | 0 | 1 | 0 | 0 | 0 | 0 | 0 | 1 | 1 | 0 | 0 | 0 | 0 |
| NM_001025575 | 0 | 0 | 0 | 0 | 0 | 0 | 0 | 0 | 0 | 0 | 0 | 0 | 0 | 1 | 1 | 0 | 0 | 0 | 1 |
| NM_134127 | 0 | 0 | 0 | 0 | 0 | 0 | 0 | 1 | 0 | 0 | 0 | 0 | 0 | 1 | 1 | 0 | 0 | 0 | 0 |
| NM_031164 | 0 | 0 | 0 | 0 | 0 | 0 | 0 | 0 | 0 | 0 | 0 | 0 | 2 | 1 | 1 | 0 | 0 | 0 | 0 |
| NM_010168 | 0 | 0 | 0 | 0 | 0 | 0 | 0 | 0 | 0 | 0 | 0 | 1 | 0 | 1 | 1 | 0 | 0 | 0 | 0 |
| NM_007818 | 0 | 0 | 0 | 0 | 0 | 0 | 0 | 0 | 0 | 0 | 0 | 1 | 0 | 1 | 2 | 0 | 0 | 0 | 0 |
| NM_008256 | 0 | 0 | 0 | 0 | 0 | 0 | 0 | 0 | 0 | 0 | 1 | 0 | 1 | 0 | 1 | 0 | 0 | 0 | 0 |
| NM_023125 | 0 | 0 | 0 | 0 | 0 | 0 | 0 | 0 | 1 | 0 | 1 | 0 | 1 | 0 | 1 | 0 | 0 | 0 | 0 |
| NM_023530 | 0 | 0 | 0 | 0 | 0 | 0 | 0 | 0 | 0 | 0 | 0 | 0 | 1 | 0 | 1 | 1 | 0 | 0 | 0 |
| NM_009243 | 0 | 0 | 0 | 0 | 0 | 0 | 0 | 0 | 0 | 0 | 0 | 1 | 1 | 0 | 1 | 2 | 0 | 0 | 0 |
| NM_009244 | 0 | 0 | 0 | 0 | 0 | 0 | 0 | 0 | 0 | 0 | 0 | 1 | 1 | 0 | 1 | 2 | 0 | 0 | 0 |
| NM_009245 | 0 | 0 | 0 | 0 | 0 | 0 | 0 | 0 | 0 | 0 | 0 | 1 | 1 | 0 | 1 | 2 | 0 | 0 | 0 |
| NM_017396 | 0 | 0 | 0 | 0 | 0 | 0 | 0 | 0 | 0 | 0 | 0 | 1 | 1 | 0 | 2 | 0 | 0 | 0 | 0 |
| NM_007822 | 0 | 0 | 0 | 0 | 0 | 0 | 0 | 1 | 0 | 0 | 0 | 0 | 1 | 0 | 1 | 0 | 0 | 0 | 0 |
| NM_007976 | 0 | 0 | 0 | 0 | 0 | 0 | 0 | 0 | 0 | 0 | 0 | 0 | 1 | 0 | 1 | 0 | 0 | 0 | 0 |
| NM_013474 | 0 | 0 | 0 | 0 | 0 | 0 | 0 | 0 | 0 | 0 | 0 | 0 | 1 | 0 | 1 | 0 | 0 | 0 | 0 |
| NM_011044 | 0 | 0 | 0 | 0 | 0 | 0 | 0 | 1 | 0 | 0 | 0 | 0 | 1 | 0 | 2 | 0 | 1 | 0 | 0 |
| NM_010172 | 0 | 0 | 0 | 0 | 0 | 0 | 0 | 0 | 0 | 0 | 0 | 0 | 0 | 0 | 1 | 0 | 1 | 0 | 0 |
| NM_023114 | 0 | 0 | 0 | 0 | 0 | 0 | 0 | 0 | 0 | 0 | 0 | 0 | 0 | 0 | 2 | 0 | 1 | 0 | 0 |
| NM_009246 | 0 | 0 | 0 | 0 | 0 | 0 | 0 | 0 | 0 | 0 | 0 | 0 | 0 | 0 | 1 | 1 | 0 | 0 | 0 |
| NM_010001 | 0 | 0 | 0 | 0 | 0 | 0 | 0 | 1 | 0 | 0 | 0 | 0 | 0 | 0 | 1 | 0 | 0 | 0 | 0 |
| NM_010012 | 0 | 0 | 0 | 0 | 0 | 0 | 0 | 0 | 0 | 0 | 0 | 0 | 0 | 0 | 1 | 0 | 0 | 0 | 0 |
| NM_206537 | 0 | 0 | 0 | 0 | 0 | 0 | 0 | 1 | 0 | 0 | 0 | 0 | 0 | 0 | 1 | 0 | 0 | 0 | 0 |
| NM_010775 | 0 | 0 | 0 | 0 | 0 | 0 | 0 | 0 | 0 | 0 | 0 | 0 | 0 | 0 | 1 | 0 | 0 | 0 | 0 |
| NM_080844 | 0 | 0 | 0 | 0 | 0 | 0 | 0 | 0 | 0 | 0 | 0 | 0 | 0 | 0 | 2 | 0 | 0 | 0 | 0 |
| NM_021489 | 0 | 0 | 0 | 0 | 0 | 0 | 0 | 0 | 0 | 0 | 0 | 0 | 0 | 0 | 1 | 0 | 0 | 0 | 0 |
| NM_028094 | 0 | 0 | 0 | 0 | 0 | 0 | 0 | 0 | 0 | 0 | 0 | 0 | 0 | 0 | 1 | 0 | 0 | 0 | 0 |
| NM_008455 | 0 | 0 | 0 | 0 | 0 | 0 | 0 | 1 | 0 | 0 | 0 | 0 | 0 | 0 | 1 | 0 | 0 | 0 | 0 |
| NM_010767 | 0 | 0 | 0 | 0 | 0 | 0 | 0 | 0 | 0 | 0 | 0 | 0 | 0 | 0 | 1 | 0 | 0 | 1 | 0 |
| NM_001003893 | 0 | 0 | 0 | 0 | 0 | 0 | 0 | 0 | 0 | 0 | 0 | 0 | 0 | 0 | 1 | 0 | 0 | 1 | 0 |
| NM_009999 | 0 | 0 | 0 | 0 | 0 | 0 | 0 | 0 | 0 | 0 | 0 | 0 | 0 | 0 | 0 | 0 | 0 | 1 | 0 |
| NM_009998 | 0 | 0 | 0 | 0 | 0 | 0 | 0 | 0 | 0 | 0 | 0 | 0 | 0 | 0 | 0 | 0 | 0 | 1 | 0 |
| NM_133862 | 0 | 0 | 0 | 0 | 0 | 0 | 0 | 0 | 0 | 0 | 0 | 0 | 0 | 0 | 0 | 0 | 0 | 0 | 1 |
| NM_019503 | 0 | 0 | 0 | 0 | 0 | 0 | 0 | 0 | 0 | 0 | 0 | 0 | 0 | 0 | 0 | 0 | 0 | 0 | 1 |
| NM_194321 | 0 | 0 | 0 | 0 | 0 | 0 | 0 | 0 | 0 | 0 | 0 | 0 | 0 | 0 | 0 | 0 | 0 | 0 | 1 |
| NM_052991 | 0 | 0 | 0 | 0 | 0 | 0 | 0 | 0 | 0 | 0 | 0 | 0 | 0 | 0 | 0 | 0 | 0 | 0 | 1 |
| NM_052992 | 0 | 0 | 0 | 0 | 0 | 0 | 0 | 0 | 0 | 0 | 0 | 0 | 0 | 0 | 0 | 0 | 0 | 0 | 1 |

**Supplementary Table 7.** Probesets and corresponding genes which have low expression in developed liver, are at least two-fold higher in LPCs and HCCs, and also follow this pattern in Oikawa *et. al.** hepatocytes (AHEP), hepatoblasts (HBs) and fibrolamellar HCCs (FLHCC). Expression data from these groups were averaged, and as per the Oikawa *et. al.* methodology, genes were retained only where expression counts were >50 in at least one of the cell categories (AHEP or HBs+FLHCC). Similarly, only probesets exceeding 256 expression units in at least one of the categories were included. Fold-changes are presented for both this study and that of Oikawa *et. al.*

| **Probeset** | **Gene Symbol (Mm)** | **FC (HCC/DL)** | **FC (LPC/DL)** | **Gene Symbol (Hs)** | **FC (FLHCC/AHEP)** | **FC (HBs/AHEP)** |
| --- | --- | --- | --- | --- | --- | --- |
| 1420907_at | Cd2ap^#^ | 15.03 | 95.14 | CD2AP | 2.73 | 2.07 |
| 1448261_at | Cdh1 | 8.89 | 4.63 | CDH1 | 5.32 | 3.12 |
| 1420326_s_at | Cramp1l | 3.44 | 5.27 | CRAMP1L | 3.44 | 3.14 |
| 1415971_at | Marcks^#^ | 6.39 | 40.19 | MARCKS | 2.01 | 2.52 |
| 1433575_at | Sox4 | 1351.34 | 30729.41 | SOX4 | 5.79 | 2.33 |

# Fold changes for these genes are the averages of two probsets, the others being 1420908_at and 1415972_at for Cd2ap and Marcks respectively.

* Oikawa, T. et al. Model of fibrolamellar hepatocellular carcinomas reveals striking enrichment in cancer stem cells. Nat. Commun. 6:8070 doi: 10.1038/ncomms9070 (2015).
